# The model structure of the copper-dependent ammonia monooxygenase

**DOI:** 10.1007/s00775-020-01820-0

**Published:** 2020-09-14

**Authors:** Francesco Musiani, Valquiria Broll, Elisa Evangelisti, Stefano Ciurli

**Affiliations:** Laboratory of Bioinorganic Chemistry, Department of Pharmacy and Biotechnology, University of Bologna, Viale G. Fanin 40, 40127 Bologna, Italy

**Keywords:** Ammonia monooxygenase, Homology modelling, Nitrogen cycle, Nitrification, Copper enzyme, *Nitrosomonas europaea*

## Abstract

**Abstract:**

Ammonia monooxygenase is a copper-dependent membrane-bound enzyme that catalyzes the first step of nitrification in ammonia-oxidizing bacteria to convert ammonia to hydroxylamine, through the reductive insertion of a dioxygen-derived O atom in an N–H bond. This reaction is analogous to that carried out by particulate methane monooxygenase, which catalyzes the conversion of methane to methanol. The enzymatic activity of ammonia monooxygenase must be modulated to reduce the release of nitrogen-based soil nutrients for crop production into the atmosphere or underground waters, a phenomenon known to significantly decrease the efficiency of primary production as well as increase air and water pollution. The structure of ammonia monooxygenase is not available, rendering the rational design of enzyme inhibitors impossible. This study describes a successful attempt to build a structural model of ammonia monooxygenase, and its accessory proteins AmoD and AmoE, from *Nitrosomonas europaea*, taking advantage of the high sequence similarity with particulate methane monooxygenase and the homologous PmoD protein, for which crystal structures are instead available. The results obtained not only provide the structural details of the proteins ternary and quaternary structures, but also suggest a location for the copper-containing active site for both ammonia and methane monooxygenases, as well as support a proposed structure of a CuA-analogue dinuclear copper site in AmoD and PmoD.

**Graphic abstract:**

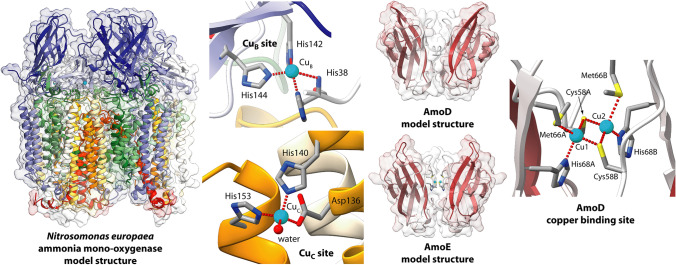

**Electronic supplementary material:**

The online version of this article (10.1007/s00775-020-01820-0) contains supplementary material, which is available to authorized users.

## Introduction

It has been estimated that the world population will reach 9 billion by the year 2050 [1], and that to sustain the consequential food demand, a 70–100% expansion in global agricultural production will be needed [2]. Nitrogen (N) is an essential element for life on Earth [3] as well as a critical nutrient for agriculture and food production [4]; due to its tremendous importance on agriculture, soil nitrogen fertilization must thus be carried out to increase crop yield [5]. According to the Food and Agriculture Organization of the United Nations (FAO), the world nitrogen fertilizer demand is expected to increase continuously for the period between 2017 and 2022 [6], and only in the United States of America (USA), nitrogen fertilizers use has increased more than 40 times from 1950 to 2015 [7].

However, concerns exist about human impact on the global N cycle [8, 9] and novel N management approaches are essential for sustainable soil fertilization and crop productivity [10]. In particular, unlike phosphorus, N possesses high reactivity in the environment and is prone to significant losses, being leached to underground water or released to the atmosphere as a product of nitrification, denitrification, leaching, and volatilization [3, 7, 8, 10–12]. Indeed, nowadays, almost 60% of N_2_O and ca. 23% of total global NO_x_ emissions come from agriculture, and the continuous increase of food demand, resulting in an increased use of nitrogen fertilizers, will contribute even more to the nitrogen gases emission in the coming years [10].

According to a report of the International Fertilizer Association (IFA), around 60% of all nitrogen fertilizers in use are based on urea [CO(NH_2_)_2_] [13], a chemical that represents 55% of the whole market [14]. Upon deposition in soil, urea is rapidly hydrolyzed to ammonium (NH_4_^+^) and bicarbonate (HCO_3_^−^), a process catalyzed by the nickel-dependent enzyme urease (urea aminohydrolase, EC 3.5.1.5) [15, 16] commonly found in soils used for crop production [14] both as intra- and extra-cellular enzyme [17]. This hydrolysis causes a rapid pH increase in the medium that leads to the formation of gaseous ammonia (NH_3_) and consequent N loss from soil.

The NH_4_^+^ ion formed upon urea hydrolysis serves as a nutrient to plants [5] as well as for aerobic respiration conducted by specific microorganisms that carry out a nitrification process that leads to the formation of nitrate (NO_3_^−^) via nitrite (NO_2_^−^). This is a mutualistic symbiosis involving ammonia-oxidizing bacteria (AOB) and Archaea (AOA), which convert ammonia to nitrite [5], and nitrite-oxidizing bacteria (NOB) that convert nitrite to nitrate [18, 19]; the entire process can also be carried out directly by ammonia-oxidizing (Comammox) bacteria [20, 21]. Nitrate thus formed in these processes can either be taken up by plant roots or enter an anaerobic denitrification route [22], being converted back to nitrite by the Mo-dependent nitrate reductase (NAR); nitrite is then transformed to gaseous forms of N such as nitric oxide (NO), nitrous oxide (N_2_O), and eventually dinitrogen (N_2_) [23], while a large portion of nitrate is also eventually leached into groundwater [5].

As a consequence of these processes, as much as 50% of nitrogen fertilizer applied to soil is not used by crops and is lost to the environment, either as gaseous species (NH_3_, NO, N_2_O, N_2_), some of which significantly contribute to the greenhouse effect [24] and the formation of air particulate matter [25], or as leached NO_3_^−^, which is a source of eutrophication [26–28]. This loss represents a very significant economic and environmental cost to farmers specifically, and for society more generally. These considerations highlight the need for the development of efficient inhibitors of nitrification.

Currently, a handful of nitrification inhibitors are used in agricultural practice. In particular, dicyandiamide (DCD), 2-chloro-6-(trichloromethyl) pyridine (Nitrapyrin), and 3,4-dimethylpyrazole phosphate (DMPP) are most frequently used [5]. However, their mode of action is not known at the molecular level, and they are thought to act as chelators of the essential copper atom present in the active site of AMO, an unproven hypothesis, while other more potent inhibitors are known but not marketed for field applications [5]. Moreover, their efficacy to reduce nitrogen losses has been shown to be highly variable and depending on many environmental conditions [29, 30]. In any case, it is important to consider the environmental toxicity, the solubility as well as the concentrations required to modulate nitrification [31, 32]. For these reasons, the search for new inhibitors is necessary to increase the efficiency of soil nitrogen fertilization toward an environmentally sustainable agriculture.

The initial step of nitrification is the oxidation of NH_4_^+^ to hydroxyl amine (NH_2_OH), catalyzed by the copper-dependent ammonia monooxygenase (AMO); this step is followed by the formation of nitrite (NO_2_^−^) catalyzed by the iron-dependent hydroxylamine oxidoreductase (HAO), and finally by the formation of nitrate (NO_3_^−^), catalyzed by the molybdenum-dependent nitrite oxidoreductase (NIX) [5]. AMO, present both in AOA and AOB (comprising both β- and γ-proteobacteria) [33] as well as in Comammox bacteria [5, 19], is thus the key enzyme to focus on for the purpose of modulating the nitrification activity in soils. In particular, *Nitrosomonas europaea* (*Ne*), a β-proteobacterium, is the most studied example of AOB [34], and *Ne*AMO, a heterotrimeric (αβγ)_3_ transmembrane copper-dependent enzyme, will be the focus of the present study.

*N. europaea* presents two nearly identical functional *amo* operon copies composed by *amo*C, *amo*A, and *amo*B (*amo*CAB), followed downstream by two open reading frames (namely Orf4 and Orf5) [18, 35]. Differently, the functional AMO operon found in γ-AOB bacteria is present only once, and while it contains Orf5, it does not comprise Orf4 [18], inducing the designation of the highly conserved Orf5 as *amo*D [36]. Orf4 (also called *amo*E) is described as a complete gene duplication of Orf5, present in all β-AOB [18, 33]. Both genes, *amo*D and *amo*E, have a highly conserved sequence and are similarly localized in the AMO operon [36], suggesting that both genes could codify for a protein playing an important role in ammonia oxidation [18]. Genomic studies on AMO are available [37], but the problems experienced in its purification as an active enzyme has significantly hampered the expansion of the structural and mechanistic knowledge on this protein in the last 30 years [34, 35].

**Scheme 1:**
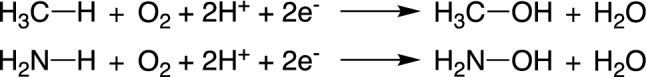
Reactions catalyzed by pMMO and by AMO

AMO features a high evolutionary correlation with particulate methane monooxygenase (pMMO), a heterotrimeric transmembrane copper-dependent enzyme that catalyzes the conversion of methane to methanol by insertion of an O_2_-derived O atom in a C–H bond [38–40], a reaction analogous to that catalyzed by AMO, which, in turn, inserts an O atom in an N–H bond, releasing a water molecule in both cases (Scheme 1).

pMMO is composed of three subunits PmoA, PmoB, and PmoC codified by the pMMO functional operon (*pmo*CAB), which is found in all methanotrophs [41]. Moreover, the pMMO and the AMO operons feature exactly the same gene structure, being *pmo*C followed by *pmo*A, *pmo*B, and *pmo*D [18, 35].

**Fig. 1:**
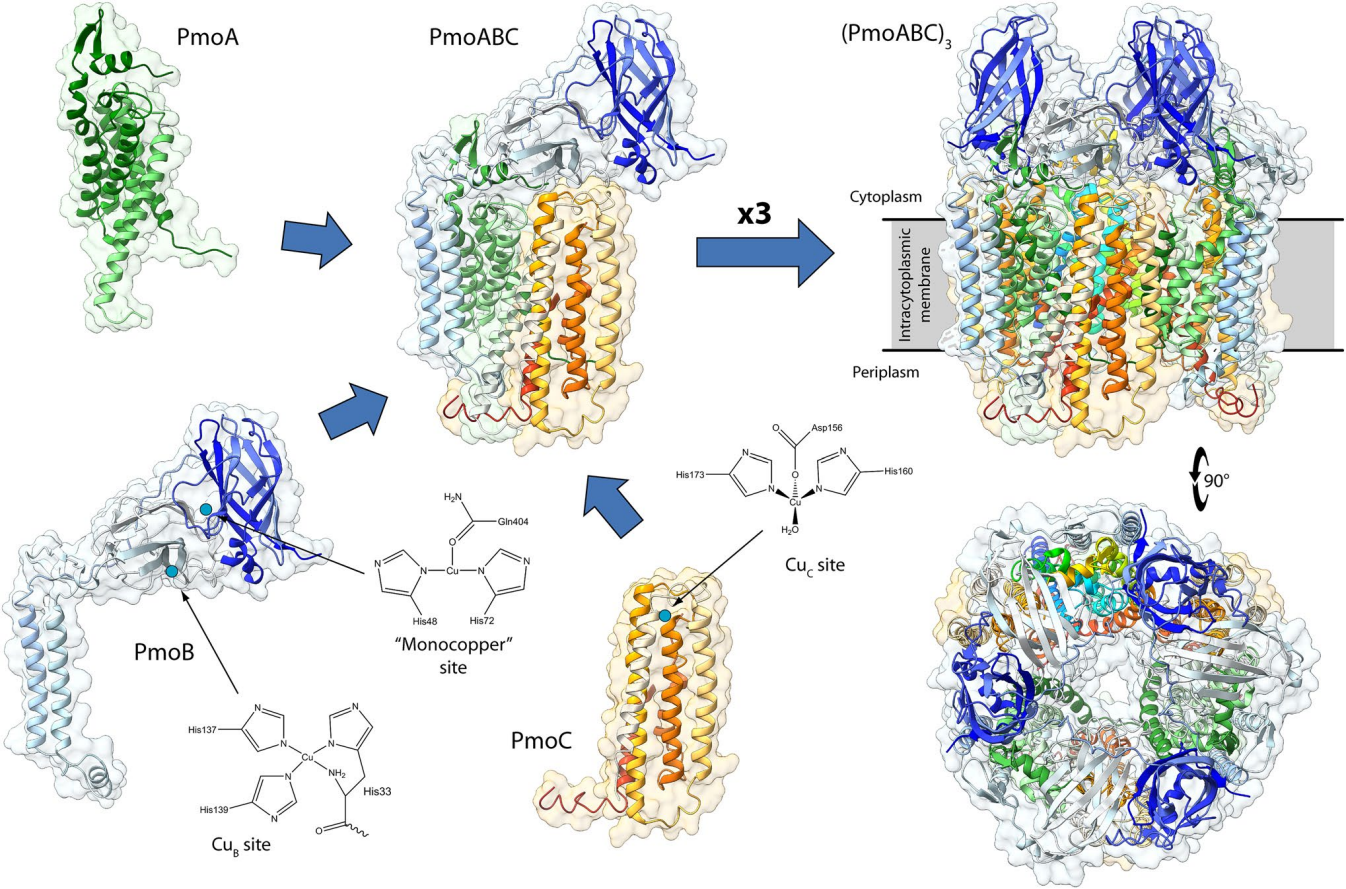
Ribbon scheme and molecular surface of *Mc*-pMMO subunits (PmoA, Pmo, and PmoC), trimer (PmoABC), and trimer of timers [(PmoABC)_3_] (PDB id 3RGB [42]). The ribbons are colored from white in correspondence of the N-terminals to dark green, dark blue, and orange in correspondence of the C-terminals for PmoA, PmoB, and PmoC, respectively. The positions (blue dots) and the schemes of the copper sites (“monocopper”, Cu_B_, and Cu_C_) are also reported. The Cu_B_ and the Cu_C_ sites have been reported accordingly to the recent literature (see Ref [48]. and [49], respectively). The orientation of the (PmoABC)_3_ in the bottom-right panel has been rotated by 90° around the horizontal axis with respect to the orientation in the upper right panel. The membrane position is indicated in the upper right panel by a gray band

The structures of pMMO from the methane-oxidizing bacteria *Methylococcus capsulatus* (strain ATCC 33,009 / NCIMB 11,132 / Bath) (*Mc*, PDB id: 1YEW, replaced by PDB id 3RGB [42]), *Methylosinus trichosporium* OB3b (*Mt*, PDB id: 3CHX), *Methylocystis* sp. strain M (*MM*, PDB id: 3RFR), *Methylocystis* sp. ATCC 49,242 (Rockwell) (*MR*, PDB id: 4PHZ, 4PI0, and 4PI2), and *Methylomicrobium alcaliphilum* 20Z (*Ma*, PDB id: 6CXH) were determined in the recent years by group of Rosenzweig [42–46] (Fig. 1). The enzyme features an homotrimer of heterotrimeric PmoABC units (PmoABC)_3_ that span the bacterial membrane (Fig. 1). Three copper-binding sites have been identified, namely the so-called “monocopper” site as well as the Cu_B_ and Cu_C_ sites. The monomeric copper site is located in the PmoB subunit bound to the Nδ atoms of His48 and His72 as well as to the carbonyl O atom of Gln404 (Fig. 1); this site has been observed only in the pMMO structure from *M. capsulatus*, while it is not conserved in pMMO’s from other bacteria [47]. On the other hand, the Cu_B_ and Cu_C_ sites are conserved in all pMMO’s so far investigated. In particular, the Cu_B_ center is located in the PmoB subunit and contains one Cu atom coordinated by the amino group and the imidazole Nδ atom of His33 together with the Nε atoms of His137 and His139 in a distorted tetrahedral geometry [48] (Fig. 1), while the Cu_C_ site is located in the PmoC subunit and appears to feature a single Cu atom bound to Asp156 Oδ, His160 Nε, His173 Nε, and a water molecule that completes a flattened tetrahedral geometry [49] (Fig. 1). Thus far, however, the crystal structures have not fully established the location and composition of the pMMO active site [48], but all evidence points to either the Cu_B_ or the Cu_C_ site for this role. It is the opinion of the authors of the present study that the latter, with its labile water-bound position, should more logically constitute the enzyme active metal site.

**Fig. 2:**
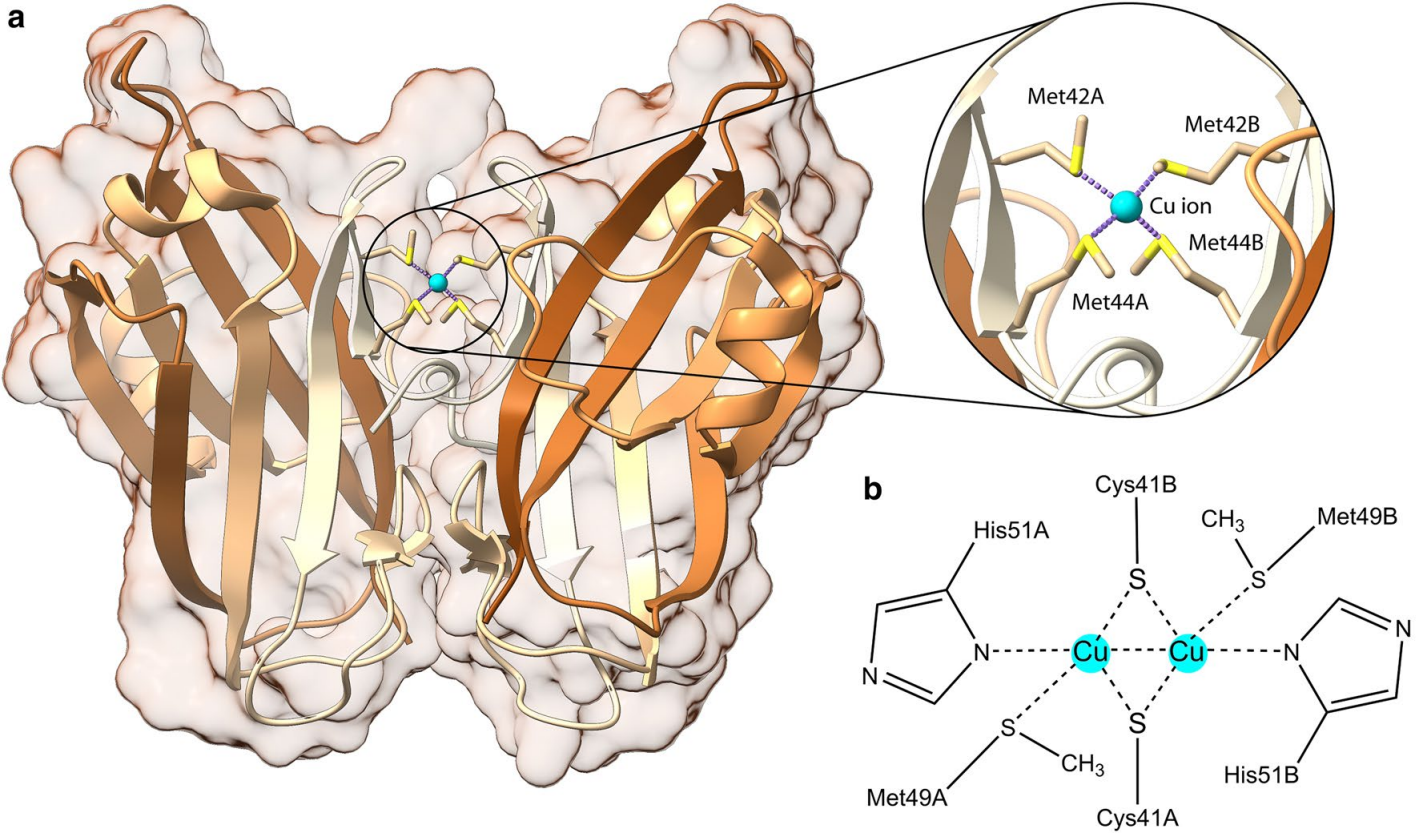
**a** Ribbon scheme and molecular surface of *Mr*PmoD (PDB id: 6CPD) [35]. The ribbons are colored from white in correspondence of the N-terminal to brown in correspondence of the C-terminal. The copper ion is represented with a cyan sphere, while the copper-binding residues are reported as sticks colored accordingly to the atom type. **b** Scheme of the proposed PmoD Cu_A_ copper site [35]

Recently, group of Rosenzweig has also determined the structure of PmoD from *Methylocystis* sp. ATCC 49,242 (Rockwell) (*Mr*PmoD, PDB id: 6CPD) [35] (Fig. 2a). PmoD, a protein encoded within many *pmo* operons, is homologous to the AmoD proteins encoded within AOB *amo* operons and has been proposed to facilitate loading, assembly, and stabilization of the active sites and/or delivery of electrons and protons to pMMO [35]. The *pmoD* gene is adjacent or close to the genes encoding for the pMMO enzyme subunits in α-, β-, and γ-proteobacterial methane-oxidizing bacteria (α-MOB, β-MOB, and γ-MOB, respectively) [35, 50]. The same occurs for the *amoD* gene in AOB (including the *Nitrosomonas*, *Nitrosospira*, and *Nitrosovibrio* genera [18]), while in β-proteobacterial *amo* operons an additional gene, homologous to *amoD* and denoted *amoE*/*orf4*, precedes *amoD*. The *Mr*PmoD sequence comprises an N-terminal signal peptide followed by a periplasmic domain containing two strictly conserved cysteine residues and a C-terminal transmembrane helix. Size exclusion chromatography coupled with multi-angle light scattering (SEC-MALS) analysis suggested that the Cu-loaded periplasmic domain is present in solution both as a monomer and as a dimer [35]. The absorption spectrum of the copper-loaded *Mr*PmoD and its dimeric form give results similar to those observed in the case of the dinuclear Cu_A_ site of the cytochrome *c* oxidase (CcO), nitrous oxide reductase (N_2_OR), and engineered Cu_A_ proteins, including Cu_A_ azurin [51], while the same features are not observed in the monomeric form [35]. The Cu_A_ center is characterized by the presence of a mixed-valence Cu(+ 1.5)–Cu(+ 1.5) site in which two copper ions separated by ca. 2.5 Å are bound to two bridging cysteine thiolate S atoms, to yield a Cu_2_S_2_ core, as well as to two histidine imidazole N atoms, a methionine thioether S atom and a backbone carbonyl O atom [52]. Unfortunately, only the monomeric form of PmoD, and not its dimeric form, could be crystallized [35], revealing the presence of a single copper atom located between the two monomers in the asymmetric unit, bound in tetrahedral geometry by two invariant Met residue S atoms from each monomer (Fig. 2a). The authors considered this site a crystallization artifact and proposed a different dinuclear Cu_A_ site for the active form of the dimeric *Mr*PmoD based on the structure of *Thermus thermophilus* cytochrome *c* oxidase (*Tt*CcO, PDB id: 2CUA [53]) (Fig. 2b).

To obtain structural information on AMO and its accessory protein, and taking advantage of the high sequence identity between pMMO and AMO [54], as well as between PmoD and AmoD/AmoE [35], the present study was undertaken, using homology modelling to predict the model structure of AMO, AmoD, and AmoE from *Nitrosomonas europaea* based on the structures of pMMO and PmoD available in the Protein Data Bank. The obtained structural models will be critical for the rationalization of the modulation of AMO activity by the currently known enzyme inhibitors as well as for the design of new strategies for the development of new and more efficient nitrification inhibitors.

## Materials and methods

### Homology modelling of AMO from *Nitrosomonas europaea*

Template searches for each of the three subunits of AMO from *Nitrosomonas europaea* (*Ne*AmoA, *Ne*AmoB, and *Ne*AmoC, UniProtKB id: Q04507, Q04508, and H2VFU7, respectively) were performed using the HHsearch method implemented in the HHpred server [55]. HHsearch accomplishes up to eight iterative PSI-BLAST [56] searches through filtered versions of the non-redundant (nr) database from NCBI. Using the final target alignment, a hidden Markov model (HMM) [57] profile is calculated. Homologous templates are identified by searching through a database containing HMMs for a representative subset of PDB sequences. HHsearch ranks the database matches based on the probability of the match to be homologous to the target sequence to distinguish homologous from non-homologous matches.

The most reliable templates were aligned with the target sequences of *Ne*AmoA, *Ne*AmoB, and *Ne*AmoC using the Promals3D server [58]. The obtained alignment was then used to calculate 100 structures using as templates the available crystal structures of *Mc*PmoA and *Ma*PmoA for *Ne*AmoA (PDB id 3RGB and 6CXH, respectively), *Mc*PmoB (PDB id 3RGB) for *Ne*AmoB, while *Mc*PmoC and *MR*PmoC (PDB id 3RGB and 4PI2, respectively) were used to model *Ne*AmoC. The Modeller 9.18 software [59] was used for all the computations. Symmetry restraints were included to grant the C_3v_ symmetry of the quaternary structure of the AMO trimer of trimers, while secondary structure restraints were used when needed accordingly to the prediction done with the PSIPRED 4.0 webserver [60, 61]. The best model was selected using the DOPE potential function built into Modeller [62]. A loop optimization routine was used to refine the regions that showed higher than average energy as calculated using the DOPE potential function. The Cu_B_ and Cu_C_ copper centers were included in the modelling following an established procedure that takes the advantage of the loop optimization routines implemented in Modeller [63–65]. The copper ions were considered always in the oxidized Cu(II) form. In particular, the van der Waals parameters for the Cu(II) ions were derived from the Zn(II) parameters included in the CHARMM22 force field [66] implemented in the Modeller v9.18 package by applying a scale factor of 1.01 calculated on the basis of the Cu(II) ionic radius. In all modelling calculations that included Cu(II) ions, constraints were imposed using a Gaussian-shaped energy potential for distances, angles, and dihedrals to correctly position the Cu(II) ions with respect to the experimentally identified ligated residues.

### Homology modelling of NeAmoE and NeAmoD

The same template search procedure followed by a multiple sequence alignment step used in the case of AMO was repeated for the modelling of *Ne*AmoE and *Ne*AmoD. The modelling procedure was identical, except for the fact that the template used here was the *Mr*PmoD dimeric structure (PDB id: 6CPD). The Cu_A_ site proposed for *Mr*PmoD was modelled in the *Ne*AmoE model structure using the same procedure used above for the AMO copper sites.

### Model analysis

The stereo-chemical quality of the final model structures was established using ProCheck [67] and the Prosa-web server [68, 69] to confirm the reliability of the model structures. The obtained molecular models and their molecular surfaces were displayed using UCSF Chimera [70] and UCSF ChimeraX [71].

## Results and discussion

### Homology modelling of AMO from *Nitrosomonas europaea*

The search for possible templates useful for the modelling of AMO from *Nitrosomonas europaea* yielded the pMMO structures listed in Table 1. In particular, the pMMO structure from *Methylococcus capsulatus* (strain ATCC 33,009 / NCIMB 11,132/Bath) (PDB id 3RGB) resulted as the best template for all the AMO subunits. On the other hand, the *Mc*-pMMO structure shows large disordered regions that have not been solved in the crystal structure (see Figs. S1–S3 in the Supplementary Information): residues 1–6, 192–222, and 246–247 in *Mc*PmoA, and residues 1–44, 225–253, and 287–298 in *Mc*AmoC are indeed absent. In the case of *Mc*PmoB, the first 32 residues at the N-terminal are missing, but this is due to a 5′ untranslated region [72] required for the correct localization of the protein, thus the functional form of the subunit has been fully solved in the crystal structure. To gain structural information on the missing regions in the *Mc-pMMO* structure, and considering the multiple sequence alignment carried out using the Promals3D server [58] (see Fig. S1–S3), the crystal structures of *Ma*PmoA (PDB id 6CXH) and *MR*PmoC (PDB id 4PI2) were included in the modelling procedure. Indeed, the *Ma*PmoA structure has only three unresolved residues at the N-terminal and at the C-terminal, while, in the case of *Mr*PmoC, the presence of one Zn(II) ion enabled the resolution of at least one part of the central region of the protein aligning with the *Mc*AmoC 205–233 portion, thus leaving only 13 residues without a template structure. To obviate to this setback and calculate a reasonable model structure for this region, secondary structure restraints based on the prediction performed using the PSIPRED 4.0 webserver [60, 61] were included in the computation (see Fig. S1–S3).

**Table 1:** *Ne*AMO putative template structures identified through the HHpred server

Sequence	Template (PDB id, chain)	Biological source [resolution (Å)]	Sequence identity	Unresolved regions/total length
*Ne*AmoA	3RGB,B/F/G	*Mc* (2.8)	50%	1–6, 192–212, 246–247 / 247
	6CXH,B/F/G	*Ma* (2.7)	48%	1–3, 245–247 / 247
	4PHZ,B/F/G	*MR* (2.6)	46%	1–8 / 252
	4PI0,B/F/G	*MR* (3.2)	46%	1–8 / 252
	4PI2,B/F/G	*MR* (3.3)	46%	1–8 / 252
	3RFR,B/F/G	*MM* (2.68)	50%	1–10 / 252
	3CHX,B/F/G	*Mt* (3.9)	47%	1–11, 250–252 / 252
*Ne*AmoB	3RGB,A/E/I	*Mc*	43%	1–32^a^ / 414
	6CXH,A/E/I	*Ma*	43%	1–32^a^ / 414
	4PHZ,A/E/I	*MR*	40%	1-28^a^, 417–420 / 420
	4PI0,A/E/I	*MR*	40%	1–28^a^, 419–420 / 420
	4PI2,A/E/I	*MR*	40%	1–28^a^, 419–420 / 420
	3RFR,A/E/I	*MM*	40%	1-28^a^, 415–419 / 419
	3CHX,A/E/I	*Mt*	39%	1–40^a^, 284–294, 318–327, 347–350, 427–431 / 431
*Ne*AmoC	3RGB,C/G/K	*Mc*	46%	1–44, 225–253, 287–289 /289
	6CXH,C/G/K	*Ma*	50%	1–89, 123–156, 193–218/ 250
	4PHZ,C/G/K	*MR*	46%	1–15, 138–165, 198–225 / 256
	4PI0,C/G/K	*MR*	46%	1–18, 200–223 / 256
	4PI2,C/G/K	*MR*	46%	1–15, 211–223 / 256
	3RFR,C/G/K	*MM*	48%	1–15, 198–225 / 256
	3CHX,C/G/K	*Mt*	46%	1–17, 177–256 / 256

The biological sources have been shortened as follows: *Methylococcus capsulatus* (strain ATCC 33,009 / NCIMB 11,132 / Bath) (*Mc*); *Methylosinus trichosporium* OB3b (*Mt*); *Methylocystis* sp. strain M (*MM*); *Methylocystis* sp. ATCC 49,242 (Rockwell) (*MR*); and *Methylomicrobium alcaliphilum* 20Z (*Ma*)

^a^5′ untranslated region [72]

The AMO metal-binding sites were modelled accordingly to the most recent findings on pMMO and on the conservation of pMMO metal-binding residues in the AMO sequence. In particular, the “monocopper” site is not conserved in pMMO nor in *Ne*AMO (Fig. S2). Moreover, of the three copper-binding residues observed for this site in the case of pMMO, only *Mc*PmoB His72 is fully conserved, while His48 is substituted with a glutamine or an asparagine and Gln404 is replaced with a serine in the *Ne*AmoB sequence. Thus, the “monocopper” site was not included in the modelling procedure. The Cu_B_ copper site is instead fully conserved both in pMMO and in AMO (Fig. S2) and was modelled considering one copper atom bound to the N-terminal amino group and to His38 Nδ, His143 Nε, and His142 Nε. The Cu–N distances (Table 2) for the AMO Cu_B_ site model were taken from the recent crystallographic refinement of the electron density enhanced with quantum–mechanical calculations carried out on the *Mc-*pMMO crystal structure [48]. The latter study suggested the presence of a mononuclear copper site in a flattened tetrahedral geometry, as confirmed by electron paramagnetic resonance (EPR) spectroscopic studies [49]. Finally, the Cu_C_ site in the AmoC subunit was modelled accordingly to the coordination geometry proposed for *Mc*PmoC [49], namely Asp156, His160, and His173 (corresponding to *Ne*AmoC Asp136, His140, and His153) in a distorted tetrahedral geometry comprising a water molecule as a fourth Cu(II) ligand. All the residues in the Cu_C_ copper site are fully conserved. The bond distances for the Cu_C_ site have been taken from model compounds [73]. The Cu oxidation state in the crystal structures remains unclear. On the basis of the quantum–mechanical structural refinements [48] and the EPR spectra [49], we opted for oxidized Cu(II) ions in all cases. As for the Zn-binding sites found in the pMMO structures, these are not conserved in the AMO sequence and thus were not included in the present modelling procedure.

**Table 2:** Distances, angles and dihedral constraints used in the modelling of Cu_B_ and Cu_C_ copper-binding sites in the AMO model structure

Constrained atoms		Distance
Cu_B_(II)-His38(N)		2.2 ± 0.1
Cu_B_(II)-His38(Nδ)		1.8 ± 0.1
Cu_B_(II)-His142(Nε)		2.1 ± 0.1
Cu_B_(II)-His144(Nε)		1.9 ± 0.1
Cu_C_(II)-Asp136(Oδ1)		2.0 ± 0.1
Cu_C_(II)-His140/153(Nε)		1.9 ± 0.1
Cu_C_(II)-Water(O)		1.9 ± 0.1
Bonded atoms	Constrained atoms	Angle
Cu(II)-His(N)	Cu(II)-His(N)-His(Cα)	109 ± 5
Cu(II)-His(Nδ)	Cu(II)-His(Nδ)-His(Cγ)	120 ± 10
	Cu(II)-His(Nδ)-His(Cε)	120 ± 10
Cu(II)-His(Nε)	Cu(II)-His(Nε)-His(Cδ)	120 ± 10
	Cu(II)-His(Nε)-His(Cε)	120 ± 10
Cu(II)-Asp(Oδ1)	Cu(II)-Asp(Oδ1)-His(Cγ)	109 ± 5
Bonded atoms	Constrained atoms	Dihedral
Cu(II)-His(Nδ)	Cu(II)-His(Nδ)-His(Cε)-His(Nε)	180 ± 10
	Cu(II)-His(Nδ)-His(Cγ)-His(Cδ)	180 ± 10
Cu(II)-His(Nε)	Cu(II)-His(Nε)-His(Cε)-His(Nδ)	180 ± 10
	Cu(II)-His(Nε)-His(Cδ)-His(Cγ)	180 ± 10

All constraints in the form “average distance ± 1 standard deviation”. Distances are in Angstroms while angles and dihedrals are in degrees

The resulting *Ne* AMO model structure was analyzed using ProCheck [67] and Prosa [68, 69], and the results are reported in Table S1 and Fig. S4, together with a comparison with the structural parameters of the main template (*Mc*-pMMO, PDB id 3RGB, resolution 2.8 Å). The quality of the model is comparable to that of the template crystal structure. As expected by a homology model, the structural parameters for the backbone are better than the template structure (in particular for the Ramachandran plot analysis), while the overall structural parameters are slightly poorer [74, 75]. The structural analysis is overall satisfactory for a relatively low-resolution model as the one presented here for AMO.

**Fig. 3:**
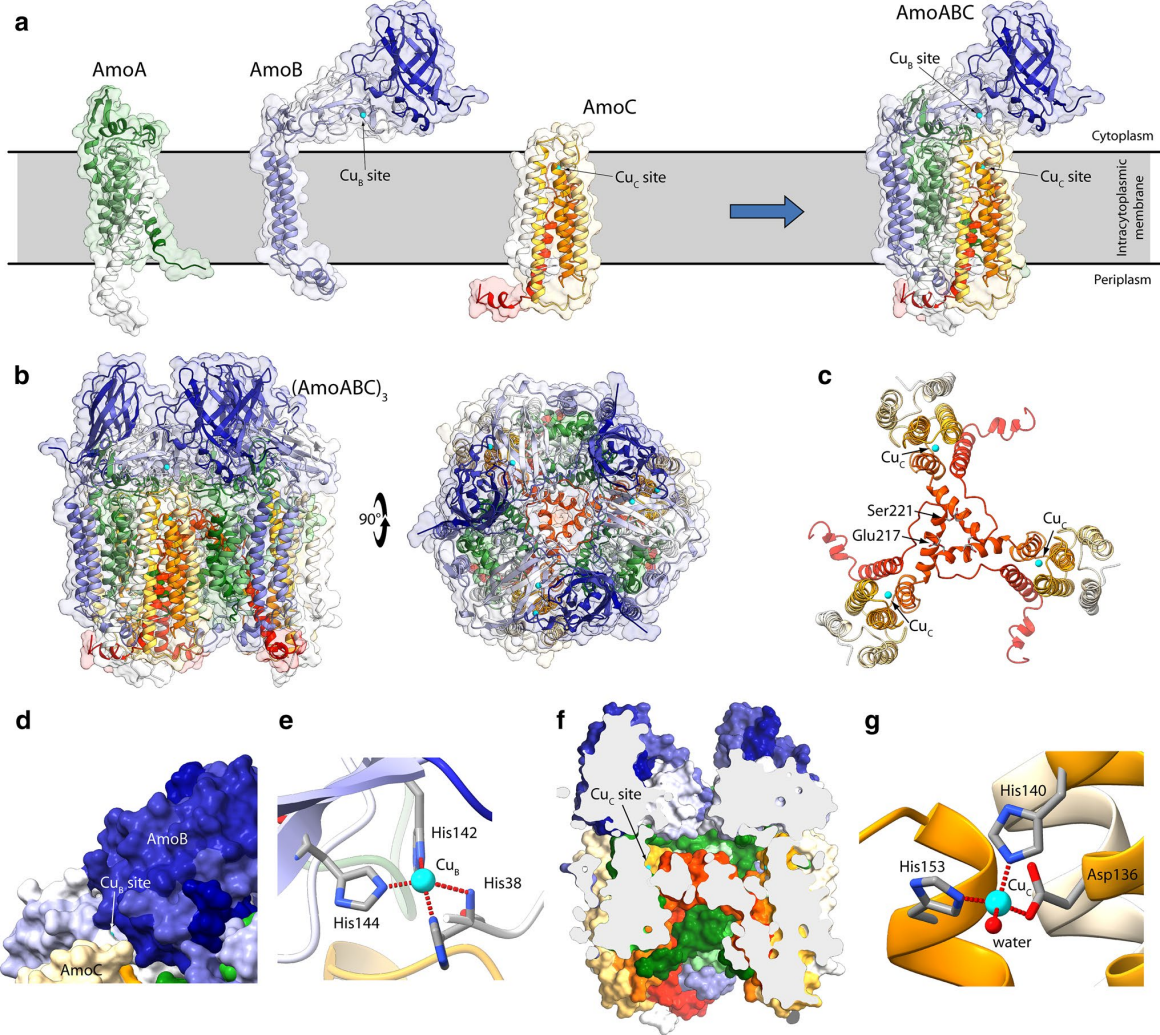
**a** Ribbon scheme and molecular surface of *Ne* AMO subunits (*Ne*AmoA, *Ne*AmoB, and *Ne*AmoC) and of the *Ne*AmoABC trimer. The position of the Cu_B_- and Cu_C_-binding sites has been shown and the Cu(II) ions are reported as cyan spheres. The ribbons and the surfaces here and in the subsequent panels are colored from white to dark green, dark blue, and red for *Ne*AmoA, *Ne*AmoB, and *Ne*AmoC respectively. The membrane position is indicated by a gray band. **b** *Ne*AmoABC trimer of trimers [(AmoABC)_3_]. The orientation in the right panel has been rotated by 90° around the horizontal axis with respect to the orientation in the left panel to show the extra-cellular side of *Ne* AMO. **c** Detail of the interaction between the three *Ne*AmoC monomers. **d** Detail of the molecular surface showing the position of the Cu_B_-binding site and the narrow tunnel at the *Ne*AmoB–*Ne*AmoC interface exposing it to the extra-cellular space. **e** Detail of the Cu_B_-binding site. **f** Longitudinal section of the *Ne*AmoABC trimer of trimers showing the large cavity found at the trimer interface and the position of the Cu_C_-binding site. **g** Detail of the Cu_C_-binding site

Figure 3a, b shows the obtained model structure of *Ne*AMO. As expected, the structure is similar to those of the template pMMO structures, with some remarkable differences. In particular, the α-helix formed by *Ne*AmoC residues 211–251 and not present in the template pMMO structure has been fully included in the *Ne*AMO model. *Ne*AmoC residues 186–200, 205–221, 231–252 were restrained to form α-helices, accordingly to the secondary structure prediction provided by the SPIPRED server [60, 61]. Interestingly, the α-helix formed by residues 205–222 is found in the center of the AMO homotrimer of heterotrimers and, together with the subsequent loop (residues 223–231), interacts with the analogous α-helix in the other *Ne*AmoC monomers (Fig. 3C). In particular, Ser221 forms an H-bond with Glu217, and van der Waals interactions are formed between residues Leu206, Trp209, Gly210, His211, Phe213, Trp214, and Glu217 from one chain and Trp214, Phe215, Glu218, Ser221, Ala222, Leu224, and Trp226 from an adjacent chain. The Cu_B_ and Cu_C_ metal-binding sites were modelled as described above and the results are reported in Fig. 3. The Cu_B_ site was found at the bottom of a narrow cleft formed by the interfaces of *Ne*AmoB and *Ne*AmoC (Fig. 3d). The Cu(II) ion was found in a distorted square planar geometry with a root-mean-square deviation (rmsd) from the ideal coordination geometry of 0.45 Å (Fig. 3e). The Cu_C_ site is instead located in a solvent-accessible cave formed by the interaction between the *Ne*AmoC α-helices at the trimer of trimers interface at about one half-height of the complex. This cave is lined on the cytoplasmic side by a surface made of the interaction of the three *Ne*AmoB subunits and is closed on the intracellular side by the *Ne*AmoC α-helices (Fig. 3f). The Cu(II) ion in the Cu_C_ site is in a slightly distorted tetrahedral geometry (rmsd from the ideal geometry = 0.14 Å, Fig. 6g).

### Homology modelling of NeAmoD and NeAmoE

**Fig. 4:**
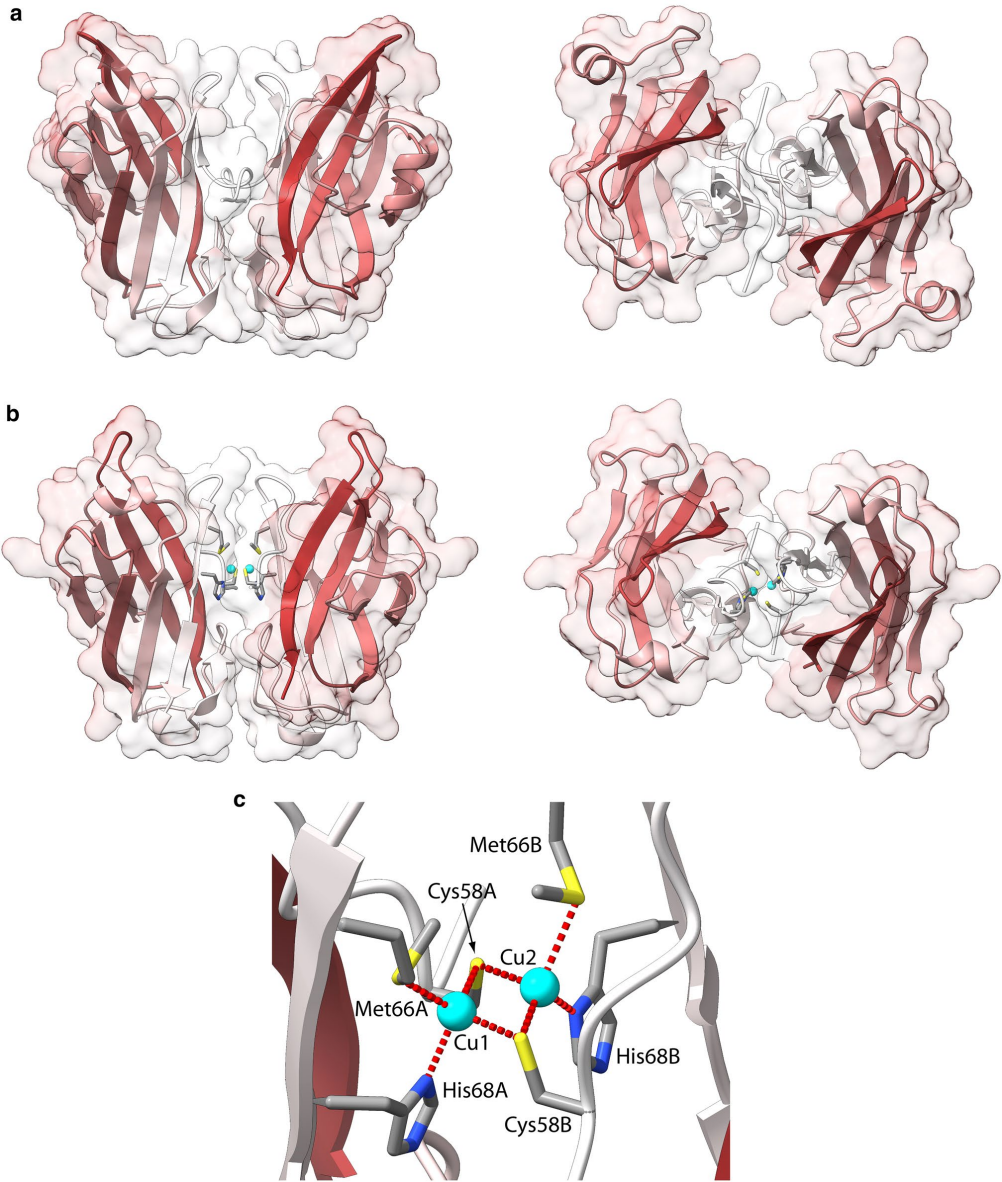
Ribbon scheme and molecular surface of *Ne*AmoD (**a**) and *Ne*AmoE (**b**) model structures. For each monomer, the ribbons are colored from white in correspondence of the N-terminal to brown in correspondence of the C-terminal. The structures in the right panels are rotated by 90° around the horizontal axis with respect to the orientation in the left panels. **c** Detail of the copper-binding site in the *Ne*AmoE model. The copper ions are represented with cyan spheres, while the copper-binding residues are reported as sticks colored accordingly to the atom type

A sequence database search for putative templates usable for the modelling of both AmoD and AmoE from *Nitrosomonas europaea* resulted in the available *Methylocystis* sp. ATCC 49,242 (Rockwell) (*Mr*PmoD, PDB id: 6CPD) [35] (Figure S5). In particular, *Ne*AmoD and *Ne*AmoE resulted to have a sequence identity of 38% and 28% with respect to *Mr*PmoD, perspectively. The Cu-binding methionine residues found in the *Mr*PmoD crystal structure are not conserved in *Ne*AmoD nor in *Ne*AmoE (Fig. S5). Instead, the proposed copper-binding residues in the dimeric form of *Mr*PmoD (Fig. 2b) are fully conserved in *Ne*AmoE and only partially conserved (two residues out of three) in *Ne*AmoD (Fig. S5). The sequence alignments with *Mr*PmoD were used to generate models of dimeric *Ne*AmoD and *Ne*AmoE in the metal free form (Fig. 4a). The copper-binding site was modelled only in the case of *Ne*AmoE, due to the complete residue conservation. The modelling was performed on the apo-*Ne*AmoE model structure using the same procedure employed for the AMO copper-binding sites and the structure of *Thermus thermophilus* CcO (*Tt*CcO, PDB id: 2CUA [53]) for the Cu-ligand distances (Table 3). The resulting *Ne*AmoD and *Ne*AmoE model structures were analyzed using ProCheck [67] and Prosa [68, 69], and the results are reported in Table S2 (and Fig. S6) and were considered fully satisfactory. As in the template structures, each monomer is composed of two antiparallel β-sheet, each composed by four β-strands, and by two short α-helices. The results of the Cu site modelling are reported in Fig. 4b, and show two Cu(II) ions (Cu1 and Cu2, hereafter) separated by 2.55 Å. The atoms of the Cu_2_S_2_ rhombus deviate from the plane by 0.2 Å, and the angle between the two CuS_2_ planes is 170.1°. Cu1 is bound to Met66A, His68A, and Cys58 from both chains in a distorted tetrahedral geometry (rmsd from ideal geometry = 0.22 Å), while Cu2 is bound to Met66B, His68B, and Cys58 from both chains, again in a distorted tetrahedral geometry (rmsd from ideal geometry = 0.30 Å). The formation of the copper complex at the *Ne*AmoE dimer interface appears to induce a conformational change of the N-terminal regions. This change appears to close the cleft formed by the loop between the first two β-strands of each monomer (see the *Ne*AmoD model structure in Fig. 4) with a consequent reduction of the protein–protein interaction surface (from 875 to 540 Å^2^ going from the apo- *Ne*AmoE to the holo- *Ne*AmoE).

**Table 3:** Distances, angles and dihedral constraints used in the modelling of copper-binding site in the *Ne*AmoE model structure

Constrained atoms		Distance
Cu1-Cys58A(Sγ)		2.30 ± 0.10
Cu1-Cys58B(Sγ)		2.30 ± 0.10
Cu2-Cys58A(Sγ)		2.30 ± 0.10
Cu2-Cys58B(Sγ)		2.30 ± 0.10
Cu1-Met66A(Sδ)		2.50 ± 0.10
Cu2-Met66B(Sδ)		2.50 ± 0.10
Cu1-His68A(Nδ),		2.10 ± 0.10
Cu2-His68B(Nδ)		2.10 ± 0.10
Cu1-Cu2		2.50 ± 0.10
Bonded atoms	Constrained atoms	Angle
Cu(II)-Cys(Sγ)	Cu(II)-Cys(Sγ)-Cys(Cβ)	109 ± 5
Cu(II)-Met(Sδ)	Cu(II)-Met(Sδ)-Met(Cβ)	109 ± 5
	Cu(II)-Met(Sδ)-Met(Cε)	109 ± 5
Cu(II)-His(Nδ)	Cu(II)-His(Nδ)-His(Cγ)	120 ± 10
	Cu(II)-His(Nδ)-His(Cε)	120 ± 10
Bonded atoms	Constrained atoms	Dihedral
Cu(II)-His(Nδ)	Cu(II)-His(Nδ)-His(Cε)-His(Nε)	180 ± 10
	Cu(II)-His(Nδ)-His(Cγ)-His(Cδ)	180 ± 10
Cu_2_S_2_	Cu1- Cys58A(Sγ)-Cu2- Cys58B(Sγ)	0 ± 10
	Cu2- Cys58B(Sγ)-Cu1- Cys58A(Sγ)	0 ± 10

All constraints in the form “average distance ± 1 standard deviation”. Distances are in Angstroms, while angles and dihedrals are in degrees

## Conclusions

The challenge to obtain the structure of the active AMO enzyme using homology modelling of the heterotrimeric enzyme and its accessory proteins AmoD and AmoE was performed based on its high sequence identity with pMMO and PmoD, respectively. The final model must, of course, be validated using experimental data possibly obtained using X-ray crystallography and/or cryo-electron microscopy. The results thus obtained provide crucial hints onto the structural framework of AMO, its quaternary, ternary, and secondary structure, as well as on the coordination environment of its metal centers. All structural findings present strong implications for its possible reaction mechanisms. Developments in this field will allow us and others to carry out the different stages of drug design and discovery that could lead to the obtainment and development of new and efficient nitrification inhibitors, decreasing nitrogen losses from soil using two different and complementary fronts, namely the main active enzyme or the accessory AmoD and AmoE proteins as a target for a virtual screening.

## Electronic supplementary material

Below is the link to the electronic supplementary material.

Supplementary file1 (PDF 419 kb)

## References

[1] Evans A (2009). The feeding of the nine billion: global food security.

[2] Godfray HCJ, Beddington JR, Crute IR, Haddad L, Lawrence D, Muir JF, Pretty J, Robinson S, Thomas SM, Toulmin C (2010). Food security: the challenge of feeding 9 billion people. Science.

[3] Canfield DE, Glazer AN, Falkowski PG (2010). The evolution and future of Earth’s nitrogen cycle. Science.

[4] Roy RN, Finck A, Blair GJ, Tandon HLS (2006). Plant nutrition for food security.

[5] Beeckman F, Motte H, Beeckman T (2018). Nitrification in agricultural soils: impact, actors and mitigation. Curr Opin Biotechnol.

[6] FAO (2017) World fertilizer trends and outlook to 2020. Food and Agriculture Organization of the United Nations

[7] Cao P, Lu C, Yu Z (2018). Historical nitrogen fertilizer use in agricultural ecosystems of the contiguous United States during 1850–2015: application rate, timing, and fertilizer types. Earth Syst Sci Data.

[8] Erisman JW, Sutton MA, Galloway J, Klimont Z, Winiwarter W (2008). How a century of ammonia synthesis changed the world. Nat Geosci.

[9] Galloway JN, Dentener FJ, Capone DG, Boyer EW, Howarth RW, Seitzinger SP, Asner GP, Cleveland CC, Green PA, Holland EA, Karl DM, Michaels AF, Porter JH, Townsend AR, Vöosmarty CJ (2004). Nitrogen cycles: past, present, and future. Biogeochemistry.

[10] Zhang X, Davidson EA, Mauzerall DL, Searchinger TD, Dumas P, Shen Y (2015). Managing nitrogen for sustainable development. Nature.

[11] Duce RA, LaRoche J, Altieri K, Arrigo KR, Baker AR, Capone DG, Cornell S, Dentener F, Galloway J, Ganeshram RS, Geider RJ, Jickells T, Kuypers MM, Langlois R, Liss PS, Liu SM, Middelburg JJ, Moore CM, Nickovic S, Oschlies A, Pedersen T, Prospero J, Schlitzer R, Seitzinger S, Sorensen LL, Uematsu M, Ulloa O, Voss M, Ward B, Zamora L (2008). Impacts of atmospheric anthropogenic nitrogen on the open ocean. Science.

[12] Oikawa PY, Ge C, Wang J, Eberwein JR, Liang LL, Allsman LA, Grantz DA, Jenerette GD (2015). Unusually high soil nitrogen oxide emissions influence air quality in a high-temperature agricultural region. Nat Commun.

[13] Prud'homme M (2016) Global fertilizer supply and trade 2016–2017.

[14] Sigurdarson JJ, Svane S, Karring H (2018). The molecular processes of urea hydrolysis in relation to ammonia emissions from agriculture. Rev Environ Sci Biotechnol.

[15] Maroney MJ, Ciurli S (2014). Nonredox nickel enzymes. Chem Rev.

[16] Mazzei L, Musiani F, Ciurli S (2017) Urease. In: Zamble D, Rowińska-Żyrek M, Kozłowski H (eds) The biological chemistry of nickel. metallobiology, vol 10. Royal Society of Chemistry, pp 60–97

[17] Burns RG (1986) Interaction of enzymes with soil mineral and organic colloids. In: Huang PMaS, M. (ed) Interactions of soil minerals with natural organics and microbes, vol Special publication n.17. Soil Science Society of America, Madison, Wisconsin, pp 429–452

[18] Arp DJ, Chain PS, Klotz MG (2007). The impact of genome analyses on our understanding of ammonia-oxidizing bacteria. Annu Rev Microbiol.

[19] Stein LY, Klotz MG (2016). The nitrogen cycle. Curr Biol.

[20] Daims H, Lebedeva EV, Pjevac P, Han P, Herbold C, Albertsen M, Jehmlich N, Palatinszky M, Vierheilig J, Bulaev A, Kirkegaard RH, von Bergen M, Rattei T, Bendinger B, Nielsen PH, Wagner M (2015). Complete nitrification by *Nitrospira bacteria*. Nature.

[21] van Kessel MAHJ, Speth DR, Albertsen M, Nielsen PH, Op den Camp HJM, Kartal B, Jetten MSM, Lücker S (2015). Complete nitrification by a single microorganism. Nature.

[22] Moura I, Maia LB, Pauleta SR, Moura JJG (2017) A bird’s eye view of denitrification in relation to the nitrogen cycle. In: Metalloenzymes in Denitrification: Applications and Environmental Impacts. The Royal Society of Chemistry, pp 1–10

[23] Maia LB, Moura JJ (2014). How biology handles nitrite. Chem Rev.

[24] Coskun D, Britto DT, Shi W, Kronzucker HJ (2017). Nitrogen transformations in modern agriculture and the role of biological nitrification inhibition. Nature Plants.

[25] Paulot F, Jacob DJ (2014). Hidden cost of US agricultural exports: particulate matter from ammonia emissions. Environ Sci Technol.

[26] Tilman D, Fargione J, Wolff B, Antonio C, Dobson A, Howarth R, Schindler D, Schlesinger WH, Simberloff D, Swackhamer D (2001). Forecasting agriculturally driven global environmental change. Science.

[27] Galloway JN, Cowling EB (2002). Reactive nitrogen and the world: 200 years of change. Ambio.

[28] Chen D, Suter H, Islam A, Edis R, Freney JR, Walker CN (2008). Prospects of improving efficiency of fertiliser nitrogen in Australian agriculture: a review of enhanced efficiency fertilisers. Aust J Soil Res.

[29] Dougherty WJ, Collins D, Van Zwieten L, Rowlings DW (2016). Nitrification (DMPP) and urease (NBPT) inhibitors had no effect on pasture yield, nitrous oxide emissions, or nitrate leaching under irrigation in a hot-dry climate. Soil Res.

[30] Friedl J, Scheer C, Rowlings DW, Deltedesco E, Gorfer M, De Rosa D, Grace PR, Muller C, Keiblinger KM (2020). Effect of the nitrification inhibitor 3,4-dimethylpyrazole phosphate (DMPP) on N-turnover, the N2O reductase-gene nosZ and N2O:N2 partitioning from agricultural soils. Sci Rep.

[31] Yang M, Fang Y, Sun D, Shi Y (2016). Efficiency of two nitrification inhibitors (dicyandiamide and 3, 4-dimethypyrazole phosphate) on soil nitrogen transformations and plant productivity: a meta-analysis. Sci Rep.

[32] Salis RK, Bruder A, Piggott JJ, Summerfield TC, Matthaei CD (2019). Multiple-stressor effects of dicyandiamide (DCD) and agricultural stressors on trait-based responses of stream benthic algal communities. Sci Total Environ.

[33] Norton JM, Alzerreca JJ, Suwa Y, Klotz MG (2002). Diversity of ammonia monooxygenase operon in autotrophic ammonia-oxidizing bacteria. Arch Microbiol.

[34] Heil J, Vereecken H, Brüggemann N (2016). A review of chemical reactions of nitrification intermediates and their role in nitrogen cycling and nitrogen trace gas formation in soil. Eur J Soil Sci.

[35] Fisher OS, Kenney GE, Ross MO, Ro SY, Lemma BE, Batelu S, Thomas PM, Sosnowski VC, DeHart CJ, Kelleher NL, Stemmler TL, Hoffman BM, Rosenzweig AC (2018). Characterization of a long overlooked copper protein from methane- and ammonia-oxidizing bacteria. Nat Commun.

[36] El Sheikh AF, Poret-Peterson AT, Klotz MG (2008). Characterization of two new genes, *amoR* and *amoD*, in the *amo* operon of the marine ammonia oxidizer *Nitrosococcus oceani* ATCC 19707. Appl Environ Microbiol.

[37] Chain P, Lamerdin J, Larimer F, Regala W, Lao V, Land M, Hauser L, Hooper A, Klotz M, Norton J, Sayavedra-Soto L, Arciero D, Hommes N, Whittaker M, Arp D (2003). Complete genome sequence of the ammonia-oxidizing bacterium and obligate chemolithoautotroph *Nitrosomonas europaea*. J Bacteriol.

[38] Rosenzweig AC (2015). Biochemistry: breaking methane. Nature.

[39] Sirajuddin S, Rosenzweig AC (2015). Enzymatic oxidation of methane. Biochemistry.

[40] Lawton TJ, Rosenzweig AC (2016). Biocatalysts for methane conversion: big progress on breaking a small substrate. Curr Opin Chem Biol.

[41] Holmes AJ, Costello A, Lidstrom ME, Murrell JC (1995). Evidence that participate methane monooxygenase and ammonia monooxygenase may be evolutionarily related. FEMS Microbiol Lett.

[42] Smith SM, Rawat S, Telser J, Hoffman BM, Stemmler TL, Rosenzweig AC (2011). Crystal structure and characterization of particulate methane monooxygenase from *Methylocystis* species strain M. Biochemistry.

[43] Lieberman RL, Rosenzweig AC (2005). Crystal structure of a membrane-bound metalloenzyme that catalyses the biological oxidation of methane. Nature.

[44] Hakemian AS, Kondapalli KC, Telser J, Hoffman BM, Stemmler TL, Rosenzweig AC (2008). The metal centers of particulate methane monooxygenase from *Methylosinus trichosporium* OB3b. Biochemistry.

[45] Sirajuddin S, Barupala D, Helling S, Marcus K, Stemmler TL, Rosenzweig AC (2014). Effects of zinc on particulate methane monooxygenase activity and structure. J Biol Chem.

[46] Ro SY, Ross MO, Deng YW, Batelu S, Lawton TJ, Hurley JD, Stemmler TL, Hoffman BM, Rosenzweig AC (2018). From micelles to bicelles: effect of the membrane on particulate methane monooxygenase activity. J Biol Chem.

[47] Balasubramanian R, Rosenzweig AC (2007). Structural and mechanistic insights into methane oxidation by particulate methane monooxygenase. Acc Chem Res.

[48] Cao L, Caldararu O, Rosenzweig AC, Ryde U (2018). Quantum refinement does not support dinuclear copper sites in crystal structures of particulate methane monooxygenase. Angew Chem Int Ed.

[49] Ross MO, MacMillan F, Wang J, Nisthal A, Lawton TJ, Olafson BD, Mayo SL, Rosenzweig AC, Hoffman BM (2019). Particulate methane monooxygenase contains only mononuclear copper centers. Science.

[50] Kenney GE, Sadek M, Rosenzweig AC (2016). Copper-responsive gene expression in the methanotroph *Methylosinus trichosporium* OB3b. Metallomics.

[51] Liu J, Chakraborty S, Hosseinzadeh P, Yu Y, Tian S, Petrik I, Bhagi A, Lu Y (2014). Metalloproteins containing cytochrome, iron-sulfur, or copper redox centers. Chem Rev.

[52] Beinert H (1997). Copper A of cytochrome c oxidase, a novel, long-embattled, biological electron-transfer site. Eur J Biochem.

[53] Williams PA, Blackburn NJ, Sanders D, Bellamy H, Stura EA, Fee JA, McRee DE (1999). The CuA domain of *Thermus thermophilus* ba3-type cytochrome c oxidase at 1.6 Å resolution. Nat Struct Biol.

[54] Shiemke AK, Arp DJ, Sayavedra-Soto LA (2004). Inhibition of membrane-bound methane monooxygenase and ammonia monooxygenase by diphenyliodonium: implications for electron transfer. J Bacteriol.

[55] Söding J, Biegert A, Lupas AN (2005). The HHpred interactive server for protein homology detection and structure prediction. Nucleic Acids Res.

[56] Altschul SF, Madden TL, Schaffer AA, Zhang J, Zhang Z, Miller W, Lipman DJ (1997). Gapped BLAST and PSI-BLAST: a new generation of protein database search programs. Nucleic Acids Res.

[57] Krogh A, Brown M, Mian IS, Sjolander K, Haussler D (1994). Hidden Markov models in computational biology. Applications to protein modeling. J Mol Biol.

[58] Pei J, Kim BH, Grishin NV (2008). PROMALS3D: a tool for multiple protein sequence and structure alignments. Nucleic Acids Res.

[59] Sali A, Blundell TL (1993). Comparative protein modelling by satisfaction of spatial restraints. J Mol Biol.

[60] Jones DT (1999). Protein secondary structure prediction based on position-specific scoring matrices. J Mol Biol.

[61] Buchan DWA, Jones DT (2019). The PSIPRED protein analysis workbench: 20 years on. Nucleic Acids Res.

[62] Shen MY, Sali A (2006). Statistical potential for assessment and prediction of protein structures. Protein Sci.

[63] Carr CE, Musiani F, Huang HT, Chivers PT, Ciurli S, Maroney MJ (2017). Glutamate ligation in the Ni(II)- and Co(II)-responsive *Escherichia coli* transcriptional regulator. RcnR Inorg Chem.

[64] Martin-Diaconescu V, Bellucci M, Musiani F, Ciurli S, Maroney MJ (2012). Unraveling the *Helicobacter pylori* UreG zinc binding site using X-ray absorption spectroscopy (XAS) and structural modeling. J Biol Inorg Chem.

[65] Barchi E, Musiani F (2019). Molecular modelling of the Ni(II)-responsive *Synechocystis* PCC 6803 transcriptional regulator InrS in the metal bound form. Inorganics.

[66] MacKerell AD, Bashford D, Bellott M, Dunbrack RL, Evanseck JD, Field MJ, Fischer S, Gao J, Guo H, Ha S, Joseph-McCarthy D, Kuchnir L, Kuczera K, Lau FT, Mattos C, Michnick S, Ngo T, Nguyen DT, Prodhom B, Reiher WE, Roux B, Schlenkrich M, Smith JC, Stote R, Straub J, Watanabe M, Wiorkiewicz-Kuczera J, Yin D, Karplus M (1998). All-atom empirical potential for molecular modeling and dynamics studies of proteins. J Phys Chem B.

[67] Laskowski RA, MacArthur MW, Moss DS, Thornton JM (1993). PROCHECK: a program to check the stereochemical quality of protein structures. J Appl Cryst.

[68] Sippl MJ (1993). Recognition of errors in three-dimensional structures of proteins. Proteins.

[69] Wiederstein M, Sippl MJ (2007) ProSA-web: interactive web service for the recognition of errors in three-dimensional structures of proteins. Nucleic Acids Res 35 (Web Server issue):W407-W410 10.1093/nar/gkm290 PMC1933241 17517781

[70] Pettersen EF, Goddard TD, Huang CC, Couch GS, Greenblatt DM, Meng EC, Ferrin TE (2004). UCSF Chimera–a visualization system for exploratory research and analysis. J Comput Chem.

[71] Goddard TD, Huang CC, Meng EC, Pettersen EF, Couch GS, Morris JH, Ferrin TE (2018). UCSF ChimeraX: meeting modern challenges in visualization and analysis. Protein Sci.

[72] Semrau JD, Chistoserdov A, Lebron J, Costello A, Davagnino J, Kenna E, Holmes AJ, Finch R, Murrell JC, Lidstrom ME (1995). Particulate methane monooxygenase genes in methanotrophs. J Bacteriol.

[73] See RF, Kruse RA, Strub WM (1998). Metal-ligand bond distances in first-row transition metal coordination compounds: Coordination number, oxidation state, and specific ligand effects. Inorg Chem.

[74] Dolan MA, Noah JW, Hurt D (2012). Comparison of common homology modeling algorithms: application of user-defined alignments. Methods Mol Biol.

[75] Dalton JA, Jackson RM (2007). An evaluation of automated homology modelling methods at low target template sequence similarity. Bioinformatics.

