# Supplementary material for: The model structure of the copper-dependent ammonia monooxygenase
Source: J Biol Inorg Chem. 2020 Sep 14;25(7):995–1007. doi: 10.1007/s00775-020-01820-0 (PMC7584546; doi:10.1007/s00775-020-01820-0)
Supplement: Supplementary file 1 — Supplementary file1 (PDF 419 kb) [file 775_2020_1820_MOESM1_ESM.pdf]

The model structure of the copper-dependent ammonia mono-oxygenase

Francesco Musiani,* Valquiria Broll, Elisa Evangelisti, Stefano Ciurli*

Laboratory of Bioinorganic Chemistry, Department of Pharmacy and Biotechnology, University of Bologna, Viale G. Fanin 40, I-40127 Bologna (Italy).

* Corresponding authors: francesco.musiani@unibo.it; stefano.ciurli@unibo.it

SUPPLEMENTARY INFORMATION

```
NeAmoA          1   MSIFRTEEILKAAKMPPEAVHMSRLIDAVYFPILIIILLVGTYHMHFMLLAGDWDFWMDWKDRQWWPVVTP   70
3RGB:B          1   MSA-----AQSAVRSHAEAVQVSRTIDWMALFVVFFVIVGSYHIHAMLTMGDWDFWSDWKDRRLWVTVTP   65
6CXH:B          1   MSA-----SQSAVRSRAEAVKVSRTFDYMILFTVFFVVLGGYHIHYMLTGGDWDFWTDWKDRRLWVTVAP   65
4PHZ:B          1   MSQSKSGGAVGPFNSVAEAAGCVQTVDWMLLVLLFFAVLGGYHVHFMLTAGDWDFWVDWKDRRMWPTVVP   70
4PI0:B          1   MSQSKSGGAVGPFNSVAEAAGCVQTVDWMLLVLLFFAVLGGYHVHFMLTAGDWDFWVDWKDRRMWPTVVP   70
4PI2:B          1   MSQSKSGGAVGPFNSVAEAAGCVQTVDWMLLVLLFFAVLGGYHVHFMLTAGDWDFWVDWKDRRMWPTVVP   70
3RFR:B          1   MSQSKSGGAVGPFNSVAEAAGCVATTDWMLLVLLFFAVLGGYHVHFMLTAGDWDFWVDWKDRRMWPTVLP   70
3CHX:B          1   MFTSKSGGAIGPFHSVAEAAGCVKTTDWMFLTLLFLAVLGGYHIHFMLTAGDWDFWVDWKDRRMWPTVVP   70
Consensus_aa:       M.......h..shp..sEAh.hsphhDhhhh.llhhhllGsYHhH@MLhtGDWDFWhDWKDRphWshVhP

NeAmoA         71   IVGITYCSAIMYYLWVNYRQPFGATLCVVCLLIGEWLTRYWGFYWWSHYPINFVTPGIMLPGALMLDFTL  140
3RGB:B         66   IVLVTFPAAVQSYLWERYRLPWGATVCVLGLLLGEWINRYFNFWGWTYFPINFVFPASLVPGAIILDTVL  135
6CXH:B         66   IVSITFPAAVQAVLWWRYRIAWGATLCVLGLLLGEWINRYFNFWGWTYFPVNFVFPSNLMPGAIVLDVIL  135
4PHZ:B         71   ILGVTFCAASQAFWWVNFRLPFGAVFAALGLLIGEWINRYVNFWGWTYFPISLVFPSALIVPAIWLDVIL  140
4PI0:B         71   ILGVTFCAASQAFWWVNFRLPFGAVFAALGLLIGEWINRYVNFWGWTYFPISLVFPSALIVPAIWLDVIL  140
4PI2:B         71   ILGVTFCAASQAFWWVNFRLPFGAVFAALGLLIGEWINRYVNFWGWTYFPISLVFPSALIVPAIWLDVIL  140
3RFR:B         71   ILGVTFCAASQAFWWVNFRLPFGAVFAVLGLMIGEWINRYVNFWGWTYFPISLVFPSAMIVPAIWLDVIL  140
3CHX:B         71   ILGVTFAAAAQAFFWENFKLPFGATFAVSGLLIGEWINRYCNFWGWTYFPISLVFPSALVVPALWLDIIM  140
Consensus_aa:       IltlT@stA.bh@hW.p@RbP@GAhhthltLLlGEWlsRYhsF@.Wo@@PIshVhPt.hlssAlhLDhhL

NeAmoA        141   YLTRNWLVTALVGGGFFGLLFYPGNWPIFGPTHLPIVVEGTLLSMADYMGHLYVRTGTPEYVRHIEQGSL  210
3RGB:B        136   MLSGSYLFTAIVGAMGWGLIFYPGNWPIIAPLHVPVEYNGMLMSIADIQGYNYVRTGTPEYIRMVEKGTL  205
6CXH:B        136   MLSNSMTLTAVVGGLAWGLLFYPGNWPIIAPLHVPVEYNGMMMTLADLQGYHYVRTGTPEYIRMVEKGTL  205
4PHZ:B        141   LLSGSYVITAVVGSLGWGLLFYPNNWPAIAAFHQATEQHGQLMTLADLIGFHFVRTSMPEYIRMVERGTL  210
4PI0:B        141   LLSGSYVITAVVGSLGWGLLFYPNNWPAIAAFHQATEQHGQLMTLADLIGFHFVRTSMPEYIRMVERGTL  210
4PI2:B        141   LLSGSYVITAVVGSLGWGLLFYPNNWPAIAAFHQATEQHGQLMTLADLIGFHFVRTSMPEYIRMVERGTL  210
3RFR:B        141   LLSGSYVITAVVGSLGWGLLFYPNNWPAIAAFHQATEQHGQLMTLADLIGLHFVRTSMPEYIRMVERGTL  210
3CHX:B        141   LLSGSYVITAVVGSLGWGLLFYPNNWPAIAALHQATEQHGQLMSLADLVGFHFVRTSMPEYIRMVERGTL  210
Consensus_aa:       hLo.s@llTAlVGt..@GLLFYPsNWPhhtshH.sh..pG.LhohADhbG@h@VRTthPEYlRhlEpGoL

NeAmoA        211   RTFGGHTTVIAAFFSAFVSMLMFTVWWYLGKVYCTAFFYVKGKRGRIVHRNDVTAFGEEGFPEGIK  276
3RGB:B        206   RTFGKDVAPVSAFFSAFMSILIYFMWHFIGRWFSNERFLQST------------------------  247
6CXH:B        206   RTFGKDVAPVSAFFSGFVSILIYFLWHFFGSWFGSEKFVQAA------------------------  247
4PHZ:B        211   RTFGKDVVPVAAFFSGFVSMMVYFLWWFMGRWYSTTKVIDTI------------------------  252
4PI0:B        211   RTFGKDVVPVAAFFSGFVSMMVYFLWWFMGRWYSTTKVIDTI------------------------  252
4PI2:B        211   RTFGKDVVPVAAFFSGFVSMMVYFLWWFMGRWYSTTKVIDTI------------------------  252
3RFR:B        211   RTFGKDVVPVAAFFSGFVSMMVYFLWWFMGRWYSTTKRIEQI------------------------  252
3CHX:B        211   RTFGKEVVPVAAFFSGFVSMMVYFLWWFVGKWYSTTKVIQKI------------------------  252
Consensus_aa:       RTFG.chhsltAFFStFVShhh@hlW@@hG+h@to.bhh.p........................
```

Figure S1. Promals3D multiple sequence alignment of *Mc*AmoA sequence with the PmoA sequence of all the pMMO structures available in the PDB (see Table 1). The secondary structure derived from PSIPRED 4.0 prediction for *Mc*AmoA sequences and from the PDB structure for PmoA is indicated (α-helix, yellow; β-strand, cyan). Residues in red and italics were not modeled due to the absence of available structural template structures. Promals3D consensus amino acid sequence symbols are also shown: conserved amino acids are in uppercase letters; aliphatic (I, V, L): l; aromatic (Y, H, W, F): @; hydrophobic (W, F, Y, M, L, I, V, A, C, T, H): h; alcohol (S, T): o; polar residues (D, E, H, K, N, Q, R, S, T): p; tiny (A, G, C, S): t; small (A, G, C, S, V, N, D, T, P): s; bulky residues (E, F, I, K, L, M, Q, R, W, Y): b; positively charged (K, R, H): +; negatively charged (D, E): -; charged (D, E, K, R, H): c.

```
NeAmoB           1  --MGIKNLYKRGVMGLYGVAYAVAALAMTVTLDVSTVAAHGERSQEPFLRMRTVQWYDIKWGPEVTKVNE   68
3RGB:A           1  MKTIKDRIAKWSA---IGLLSAVAATAF----YAPSASAHGEKSQAAFMRMRTIHWYDLSWSKEKVKINE   63
6CXH:A           1  MKIIKDKVAKLSF---VALLVTVTAAMF----YTPTASAHGEKSQAAFMRMRTIHWFDLNWSKDQVSVNE   63
4PHZ:A           1  ----MKKLVKLAA---FGAAAAVAATLG----AVAPASAHGEKSQQAFLRMRTLNWYDVQWSKTTVNVNE   59
4PI0:A           1  ----MKKLVKLAA---FGAAAAVAATLG----AVAPASAHGEKSQQAFLRMRTLNWYDVQWSKTTVNVNE   59
4PI2:A           1  ----MKKLVKLAA---FGAAAAVAATLG----AVAPASAHGEKSQQAFLRMRTLNWYDVQWSKTTVNVNE   59
3RFR:A           1  ----MKKLVKLAA---FGAAAAVAATLG----AIAPASAHGEKSQQAFLRMRTLNWYDVQWSKTTVNVNE   59
3CHX:A           1  --------------------------------------HGEKSQQAFLRMRTLNWYDVKWSKTSLNVNE   31
Consensus_aa:       ......plhK.th...hGhhhAVAAhh......hsshtAHGE+SQ.sFhRMRTlpWYDlpWt.p.hpVNE

NeAmoB          69  NAKITGKFHLAEDWPRAAAQPDFSFFNVGSPSPVFVRLSTKINGHPWFISGPLQIGRDYEFEVNLRARIP  138
3RGB:A          64  TVEIKGKFHVFEGWPETVDEPDVAFLNVGMPGPVFIRKESYIGGQLVPRSVRLEIGKTYDFRVVLKARRP  133
6CXH:A          64  TMSISGKFHVFAGWPETVDKPEVAFLNIGIPGPVFIRAGSWIGGQLVPRSVSLELGETYEFKVLLKARRP  133
4PHZ:A          60  EMVLSGKVHVFSAWPQAVANPRVSFLNAGEPGPVLVRTAQFIGEQFAPRSVSLEIGKDYAFSINLRGRRA  129
4PI0:A          60  EMVLSGKVHVFSAWPQAVANPRVSFLNAGEPGPVLVRTAQFIGEQFAPRSVSLEIGKDYAFSINLRGRRA  129
4PI2:A          60  EMVLSGKVHVFSAWPQAVANPRVSFLNAGEPGPVLVRTAQFIGEQFAPRSVSLEIGKDYAFSINLRGRRA  129
3RFR:A          60  EMILSGKVHVFSAWPQAVANPRVSFLNAGEPGPVLVRTAQFIGEQFAPRSVSLEIGKDYAFSINLRGRRA  129
3CHX:A          32  SMVLSGKVHVFSAWPQAVANPKSSFLNAGEPGPVLVRTAQFIGEQFAPRSVSLEVGKDYAFSIDLKARRA  101
Consensus_aa:       ph.loGKhHlhpsWPphhspPchtFhNhG.PtPVhlRhtpbIs.p.h..SssLplG+sY.FplsL+tR.s

NeAmoB         139  GRHHMHAMLNVKDAGPIAGPGAWMNITGSWDDFTNPLKLLTGETIDSETFNLSNGIFWHVVWMSIGIFWI  208
3RGB:A         134  GDWHVHTMMNVQGGGPIIGPGKWITVEGSMSEFRNPVTTLTGQTVDLENYNEGNTYFWHAFWFAIGVAWI  203
6CXH:A         134  GDWHVHTMMNVQGGGPIIGPGKWVTITGSMGDFKNPITTLTGETIDLETYALDGVYGWHLFWYLLGVAWM  203
4PHZ:A         130  GRWHVHAQINVEGGGPIIGPGQWIEIKGDMKDFTDPVTLLDGSTVDLEHYGISRVYAWHLPWMAVGAAWI  199
4PI0:A         130  GRWHVHAQINVEGGGPIIGPGQWIEIKGDMKDFTDPVTLLDGSTVDLEHYGISRVYAWHLPWMAVGAAWI  199
4PI2:A         130  GRWHVHAQINVEGGGPIIGPGQWIEIKGDMKDFTDPVTLLDGSTVDLEHYGISRVYAWHLPWMAVGAAWI  199
3RFR:A         130  GRWHVHAQINVEGGGPIIGPGQWIEIKGDMKDFTDPVTLLDGSTVDLENYGISRIYAWHLPWLAVGAAWI  199
3CHX:A         102  GRWHVHAQINVEGGGPIIGPGQWIEIKGDMADFKDPVTLLDGTTVDLETYGIDRIYAWHFPWMIAAAAWI  171
Consensus_aa:       Gc@HhHhbhNVpstGPIhGPG.WhpIpGsh.DFpsPlphLsGpTlD.Ep@slspshhWHh.Wh.lGhhWI

NeAmoB         209  GVFTARPMFLPRSRVLLAYGDDLLMDPMDKKITWVLAILTLALVWGGYRYTENKHPYTVPIQAGQSKV-A  277
3RGB:A         204  GYWSRRPIFIPRLLMVDAGRADELVSATDRKVAMGFLAATILIVVMAMSSANSKYPITIPLQAGTMRGMK  273
6CXH:A         204  VYWCRKPVFIPRRIAVDAGKADSLITPTDKKVGMAFAAGTLAIVAVSMGQANEKYPVTTPLQAGLMRGIK  273
4PHZ:A         200  FFWFVRKGIITSYIRVAEGKADDVIGDDDRRIGAIVLALTILATIVGYAVTNSTFPRTIPLQAGLQKPLT  269
4PI0:A         200  FFWFVRKGIITSYIRVAEGKADDVIGDDDRRIGAIVLALTILATIVGYAVTNSTFPRTIPLQAGLQKPLT  269
4PI2:A         200  FFWFVRKGIITSYIRVAEGKADDVIGDDDRRIGAIVLALTILATIVGYAVTNSTFPRTIPLQAGLQKPLT  269
3RFR:A         200  LFWFIRKGIIASYVRVAEGRPDDVIGDDDRRIGAIVLALTILATIVGYAVTNSTFPRTIPLQAGLQKPLT  269
3CHX:A         172  LYWFFKKGIIASYLRISEGKDEEQIGDDDRRVGAIVLAVTILATIIGYAVTNSTFPRTIPLQAGLQKPLT  241
Consensus_aa:       .h@h.+..hlsp...l....sD.lhss.D++lshhhhhhTlhhhh.th..hpsp@P.TlPlQAGb.+sh.

NeAmoB         278  AL------PVAPNPVSIVITDANYDVPGRALRVTMEVTNNGDIPVTFGEFTTAGIRFINSTGRKYLDPQY  341
3RGB:A         274  PL------ELPAPTVSVKVEDATYRVPGRAMRMKLTITNHGNSPIRLGEFYTASVRFLDSDVYKD-TTGY  336
6CXH:A         274  SL------ELPQPTVSVKVVDASYRVPGRAMQMTLEITNNGDSAVRLAEFNTASVRFLDADVYED-DTNY  336
4PHZ:A         270  PIETEGTVGVGKENVTTELNGGVYKVPGRELTINVKVKNNTSQPLRLGEYTAAGLRFLNPDVFTT-KPDF  338
4PI0:A         270  PIETEGTVGVGKENVTTELNGGVYKVPGRELTINVKVKNNTSQPLRLGEYTAAGLRFLNPDVFTT-KPDF  338
4PI2:A         270  PIETEGTVGVGKENVTTELNGGVYKVPGRELTINVKVKNNTSQPLRLGEYTAAGLRFLNPDVFTT-KPDF  338
3RFR:A         270  PIETEGTVGVGKEQVTTELNGGVYKVPGRELTINVKVKNGTSQPVRLGEYTAAGLRFLNPTVFTQ-KPDF  338
3CHX:A         242  PIIEEGTAGVGPHVVTAELKGGVYKVPGRELTIQVKVTNKTDEPLKLGEYTAAGLRFLNPDVFTT-KPEF  310
Consensus_aa:       sl.......ls..sVoh.lpstsYcVPGR.hphphclpNpss.PlphGE@hhAtlRFlssss.p..csp@

NeAmoB         342  PRELIAV-GLNFDDESAIQPGQTKELKMEAKDALWEIQRLMALLGDPESRFGGLLMSWDAEGNRHINSIA  410
3RGB:A         337  PEDLLAEDGLSVSDNSPLAPGETRTVDVTASDAAWEVYRLSDIIYDPDSRFAGLLFFFDATGNRQVVQID  406
6CXH:A         337  PDDLLAEEGLSVSDNSPLAPGETRTVDVTASDAAWEVYRLADLIYDPDSRFAGLLFFIDEDGNRQMTMVD  406
4PHZ:A         339  PDYLLADRGLSV-DATPIAPGEAKEIVVKIQDARWDIERLSDLAYDTDSQIGGLLFFFSPDGKRYASEIG  407
4PI0:A         339  PDYLLADRGLSV-DATPIAPGEAKEIVVKIQDARWDIERLSDLAYDTDSQIGGLLFFFSPDGKRYASEIG  407
4PI2:A         339  PDYLLADRGLSV-DATPIAPGEAKEIVVKIQDARWDIERLSDLAYDTDSQIGGLLFFFSPDGKRYASEIG  407
3RFR:A         339  PDYLLADRGLSN-DDV-IAPGESKEIVVKIQDARWDIERLSDLAYDTDSQVGGLLFFFTPDGKRFAAEIG  406
3CHX:A         311  PDYLLADRGLST-DPTPLAPGETKTIEVKVQDARWDIERLSDLAYDTDSQIGGLLMFFSPSGKRYATEIG  379
Consensus_aa:       Pc.LlA.cGLsh.D.osl.PGph+pl.hphpDA.W-lbRL.sLh.Ds-SphtGLLh.@sspGpR.hspIs

NeAmoB         411  GPVIPVFTKL---  420
3RGB:A         407  APLIPSFM-----  414
6CXH:A         407  APLIPTFI-----  414
4PHZ:A         408  GPVIPKFVAGDMP  420
4PI0:A         408  GPVIPKFVAGDMP  420
4PI2:A         408  GPVIPKFVAGDMP  420
3RFR:A         407  GPVIPKFVAGDMP  419
3CHX:A         380  GPVIPKFVAGDMP  392
Consensus_aa:       tPlIP.Fh.....
```

Figure S2. Promals3D multiple sequence alignment of *Mc*AmoB sequence with the PmoB sequence of all the pMMO structures available in the PDB (see Table 1). See Figure S1 caption for the color scheme and for the Promals3D consensus amino acid sequence symbols. Copper binding residues of the monomeric and of the Cu_B copper sites found in *Mc*AmoB have been highlighted in red and blue, respectively.

```
NeAmoC           1   -----------------------------MATTLGTSSASSVSSRGYDM-SLWYDSKFYKFGMITMLLVA   40
3RGB:C           1   MHETKQGGEKRFTGAICRCSHRYNSMEVKMAAT-----TIG-GAAAAEA-PL-LDKKWLTFALAIYTVFY   62
6CXH:C           1   -----------------------------MAAT-----TESVKADAAEA-PL-LNKKNIIAGASLYLVFY   34
4PHZ:C           1   -----------------------------MSST-----TSAAAGAAAEVESV-VDLRGMWIGLVLLNVFY   35
4PI0:C           1   -----------------------------MSST-----TSAAAGAAAEVESV-VDLRGMWIGLVLLNVFY   35
4PI2:C           1   -----------------------------MSST-----TSAAAGAAAEVESV-VDLRGMWIGLVLLNVFY   35
3RFR:C           1   -----------------------------MSST-----TSTAAGAAAEVESV-VDLRGMWIGLAVLNVFY   35
3CHX:C           1   -----------------------------MSVT-----TETTAGAAAGSDAI-VDLRGMWVGVAGLNIFY   35
Consensus_aa:        .............................MtsT.....hpthtt.th-h.sl.hD.+.hbhGhhhh.lhh

NeAmoC          41   IFWVWYQRYFAYSHGMDSMEPEFDRVWMGLWRVHMAIMPLFALVTWGWILKTRDTKEQLDNLDPKLEIKR  110
3RGB:C          63   LWVRWYEGVYGWSAGLDSFAPEFETYWMNFLYTEIVLEIVTASILWGYLWKTRD--RNLAALTPREELRR  130
6CXH:C          35   AWVRWYEGVYGWSAGLDSFAPEFETYWMNFLYIEMVLEVLTASVLWGYIWKSRD--RKVMSITPREELRR  102
4PHZ:C          36   LIVRIYEQVFGWRAGLDSFAPEFQTYWMSILWTEIPLELVSGLGLAGYLWKTRD--RNVDAVTPREEMRR  103
4PI0:C          36   LIVRIYEQVFGWRAGLDSFAPEFQTYWMSILWTEIPLELVSGLGLAGYLWKTRD--RNVDAVTPREEMRR  103
4PI2:C          36   LIVRIYEQVFGWRAGLDSFAPEFQTYWMSILWTEIPLELVSGLGLAGYLWKTRD--RNVDAVTPREEMRR  103
3RFR:C          36   LIVRIYEQVFGWRAGLDSFAPEFQTYWMSILWTEIPLELVSGLGLAGYLWKTRD--RNVDAVAPREEMRR  103
3CHX:C          36   LIVRIYEQIYGWRAGLDSFAPEFQTYWLSILWTEIPLELVSGLALAGWLWKTRD--RNVDAVAPREELRR  103
Consensus_aa:        lhh.hYp.h@t@phGhDSh.PEFpphWMshh.hchslb.l.t.shhG@lhKTRD..cplsslsP+bEh+R

NeAmoC         111   YFYYMMWLGVYIFGVYWGGSFFTEQDASWHQVIIRDTSFTPSHVVVFYGSFPMYIVCGVATYLYAMTRLP  180
3RGB:C         131   NFTHLVWLVAYAWAIYWGASYFTEQDGTWHQTIVRDTDFTPSHIIEFYLSYPIYIITGFAAFIYAKTRLP  200
6CXH:C         103   HFTHWTWLMMYGIAIYFGASYFTEQDGTWHQTIVRDTDFTPSHIIEFYLSYPIYIITGGASFLYAKTRLP  172
4PHZ:C         104   LVVLVQWLVVYGIAIYWGASFFTEQDGTWHMTVIRDTDFTPSHIIEFYMSYPIYSVIAVGAFFYAKTRIP  173
4PI0:C         104   LVVLVQWLVVYGIAIYWGASFFTEQDGTWHMTVIRDTDFTPSHIIEFYMSYPIYSVIAVGAFFYAKTRIP  173
4PI2:C         104   LVVLVQWLVVYGIAIYWGASFFTEQDGTWHMTVIRDTDFTPSHIIEFYMSYPIYSVIAVGAFFYAKTRIP  173
3RFR:C         104   LVVLVQWLVVYGIAIYWGASFFTEQDGAWHMTVIRDTDFTPSHIIEFYMSYPIYSVIAVGAFFYAKTRIP  173
3CHX:C         104   HVVLVEWLVVYAVAIYWGASFFTEQDGTWHMTVIRDTDFTPSHIIEFYMSYPIYSIMAVGAFFYAKTRIP  173
Consensus_aa:        hhhhh.WLshY.htlYWGtS@FTEQDtoWHbhllRDTsFTPSHll.FY.S@PhY.lhthth@hYAbTRlP

NeAmoC         181   LFSRGISFPLVMAIAGPLMILPNVGLNEWGHAFWFMEELFSAPLHWGFVVLGWAGLFQGGVAAQIITRYS  250
3RGB:C         201   FFAKGISLPYLVLVVGPFMILPNVGLNEWGHTFWFMEELFVAPLHYGFVIFGWLALAVMGTLTQTFYSFA  270
6CXH:C         173   TYQQGLSLQYLVVVVGPFMILPNVGLNEWGHTFWFMEELFVAPLHYGFVFFGWSALGVLGVINIELGALS  242
4PHZ:C         174   YFAHGYSLAFLIVAIGPFMIIPNVGLNEWGHTFWFMEELFVAPLHWGFVFFGWMALGVFGVVLQILMRIH  243
4PI0:C         174   YFAHGYSLAFLIVAIGPFMIIPNVGLNEWGHTFWFMEELFVAPLHWGFVFFGWMALGVFGVVLQILMRIH  243
4PI2:C         174   YFAHGYSLAFLIVAIGPFMIIPNVGLNEWGHTFWFMEELFVAPLHWGFVFFGWMALGVFGVVLQILMRIH  243
3RFR:C         174   YFAHGYSLAFLIVAIGPFMIIPNVGLNEWGHTFWFMEELFVAPLHWGFVFFGWMALGVFGVVLQILGRIH  243
3CHX:C         174   YFAHGFSLAFLIVAIGPFMIIPNVGLNEWGHTFWFMEELFVAPLHWGFVFFGWMALGVFGVVLQILMGVK  243
Consensus_aa:        hFt+GhShshlhhhhGPhMIlPNVGLNEWGHhFWFMEELFsAPLH@GFVhhGWhtL...GVhhQhl..hp

NeAmoC         251   NLTDVVWNNQSKEILNNRIVA  271
3RGB:C         271   QGGLGQSLCEAVDEGLIAK--  289
6CXH:C         243   KLLKKDLA-------------  250
4PHZ:C         244   ALVGKEGVKLLTE--------  256
4PI0:C         244   ALVGKEGVKLLTE--------  256
4PI2:C         244   ALVGKEGVKLLTE--------  256
3RFR:C         244   ALIGKEGVALLTE--------  256
3CHX:C         244   RLIGKDCVAALVG--------  256
Consensus_aa:        .....................
```

Figure S3. Promals3D multiple sequence alignment of *Mc*AmoC sequence with the PmoC sequence of all the pMMO structures available in the PDB (see Table 1). See Figure S1 caption for the color scheme and for the Promals3D consensus amino acid sequence symbols. Copper binding residues in the Cu_C copper sites found in *Mc*AmoC have been highlighted in dark greed.

Table S1. Results of the Procheck and Prosa analysis done on the *Ne* AMO model structure and on the *Mc* pMMO structure (PDB id 3RGB).

	Procheck Ramachandran plot					
**Protein**	**Most favored**	**Additionally allowed**	**Generously allowed**	**Disallowed**	**Procheck G-factor**	**Prosa Z-score**
AMO (global)	92.6%	7.4%	0.0%	0.0%	-0.28	-
AmoA	95.1%	4.1%	0.0%	0.0%	-0.23	-1.90
AmoB	91.0%	9.0%	0.0%	0.0%	-0.39	-4.04
AmoC	94.4%	5.6%	0.0%	0.0%	-0.26	-2.24
pMMO (global)	82.3%	15.2%	1.2%	1.2%	-0.17	-
PmoA	86.2%	13.3%	0.5%	0.0%	-0.13	-2.48
PmoB	81.5%	16.0%	1.2%	1.2%	-0.23	-6.29
PmoC	81.3%	13.9%	2.1%	2.7%	-0.20	-1.71

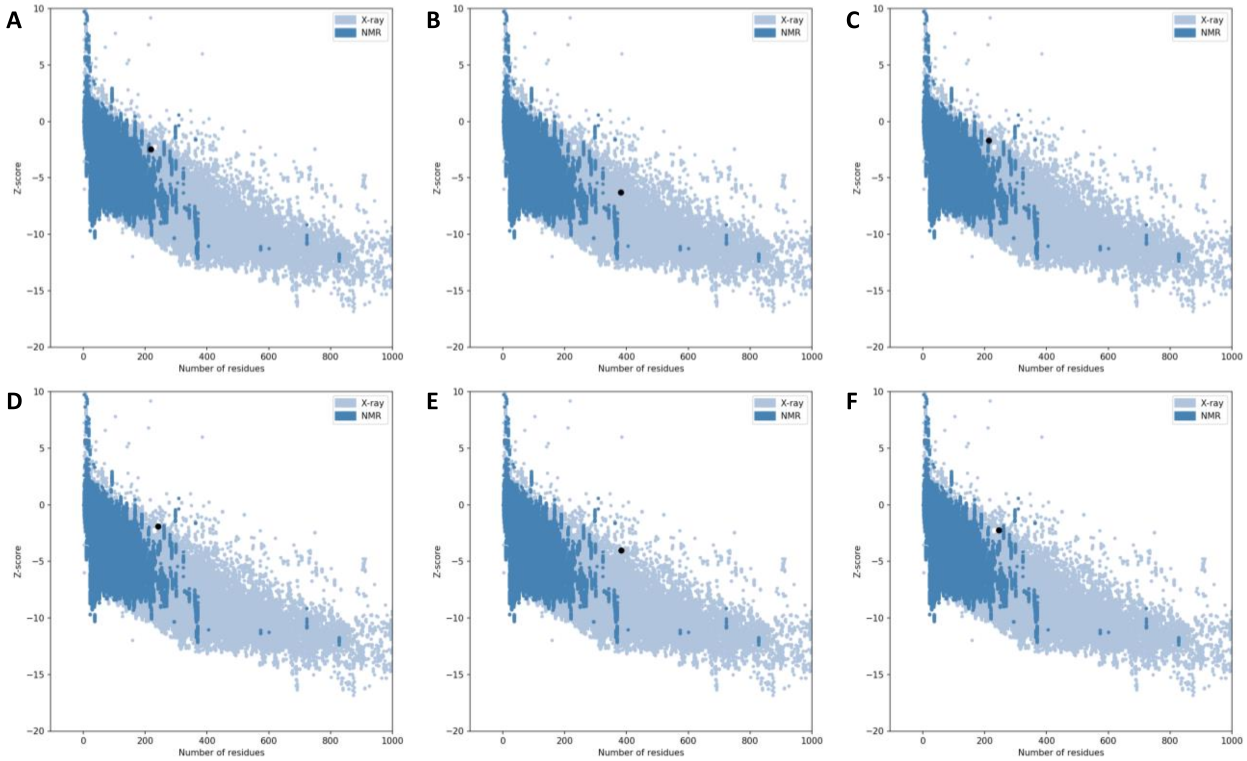

Figure S4. Prosa overall model quality plots for the *Mc*PmoA, *Mc*PmoB and *Mc*PmoB crystal structures found in the *Mc* pMMO trimer or trimers (PDB id: 3RGB) (panels **A**, **B**, and **C**, respectively) and for the *Ne*AmoA, *Ne*AmoB and *Ne*AmoC model structures generated in this work (panels **D**, **E**, and **F**, respectively).

A

```
                                   37   40         50         60      67
                                     \   |          |          |       |
MrPmoD    ----------------------------MGNMCMVMFGYDMIHITVFQPDKS-RSEYCDE
                                          **    *    * *   **      * *
NeAmoD    MTSIKWLGGVLLGMVCSIQVQAHGGLSLAEDMCKLTIGPYTMHFTGYQPESTQEKEFCED
          |        |         |         |         |         |         |
          1        10        20        30        40        50        60

       68   70          80         90         100        110        120     127
         \   |           |          |          |          |          |       |
MrPmoD    IPATGRTIMAFDIENPAFRDLPLELRIIRDPLTPVLPTGEKELDALTELHLPAKKYSKGT
          **  **** * *     * *     * *****     *  *  ** *   * * * *  *
NeAmoD    IPNIGRTIVALDYIDEALRTMTTEVRIIRDTGAE--PGSEGNLDELTVFHSPPKVYMNGS
          |        |         |         |           |         |       |
          61       70        80        90          100       110     118

      128  130        140        150        160   163
         \   |          |          |          |     /
MrPmoD    FSVEHNFANNGHYIGLVTLTRESGQQETAQFKFMVG--------------------------------
             ** *    *    ****      *      * * **
NeAmoD    VTFEHDFPAEGKFVGLVTIR-DNGTEHISRFPFAVGTGGKPDMLYILGALALAAGAGIFFFKKKQNL
         / |         |          |          |          |         |         |   |
       119 120       130        140        150        160       170       180 184
```

B

```
                                                                   37   40 43
                                                                     \   |  |
MrPmoD    -----------------------------------------------------MGNMCMV
                                                                   *
NeAmoE    MRRESLLKKHEGVVKSTGGGVVLKQSLYSIVTAFVFVILCSASTVWGHGRVSLEEDNCVR
          |        |         |         |         |         |         |
          1        10        20        30        40        50        60

              50         60          70         80         90        100   102
               |          |           |          |          |          |   /
MrPmoD    MFGYDMIHITVFQPDKS-RSEYCDEIPATGRTIMAFDIENPAFRDLPLELRIIRDPLTPV
            *  * *     **       ** **** * *     *    ** *   *      *
NeAmoE    QVGENMVHLNTYQPQYDQAGHYCTEIPAAGDTYLVVDLIDPALRNMPVSMKVFRGEEK--
          |        |         |         |           |         |     |
          61       70        80        90          100       110   116

          103    110        120        130        140        150       160   162
           |      |          |          |          |          |         |   /
MrPmoD    LPTGEKELDALTELHLPAKKYSKGTFSVEHNFANNGHYIGLVTLTRESGQQETAQFKFMV
                       *   *     *          * *   **                 *
NeAmoE    --------GGEAILQVKADYHPDGVINGIGKLD-KGLYSVMVTAEGVP--PLNYYYQLRV
                 /   |         |         |           |           |   |
               117   120       130       140         150         160 165

          163
           |
MrPmoD    G-------------------------------------------

NeAmoE    EMVDYGKLVRTWAGPAVAILFLGWLMYKLVQSGRLRSWFKSQDD
          |   |         |         |         |        |
          166 170       180       190       200      209
```

Figure S5. Promals3D multiple sequence alignment of *Mr*PmoD sequence with the *Ne*PmoD (**A**) and *Ne*PmoE (**B**) sequences. The secondary structure derived from the PDB structure for *Mr*PmoD and from PSIPRED 4.0 prediction for *Ne*PmoD and *Ne*PmoE sequences is indicated (α-helix, yellow; β-strand, cyan). Residues in red and italics were not modeled due to the absence of available structural template structures. Residues in white and highlighted in blue are those proposed for copper binding.

Table S2. Results of the Procheck and Prosa analysis done on the *Ne*AmoD and *Ne*AmoE model structures.

	Procheck Ramachandran plot					
**Protein**	**Most favored**	**Additionally allowed**	**Generously allowed**	**Disallowed**	**Procheck G-factor**	**Prosa Z-score**
*Ne*AmoD	95.2%	4.8%	0.0%	0.0%	-0.17	-6.30
*Ne*AmoE	93.7%	6.3%	0.0%	0.0%	-0.21	-4.92

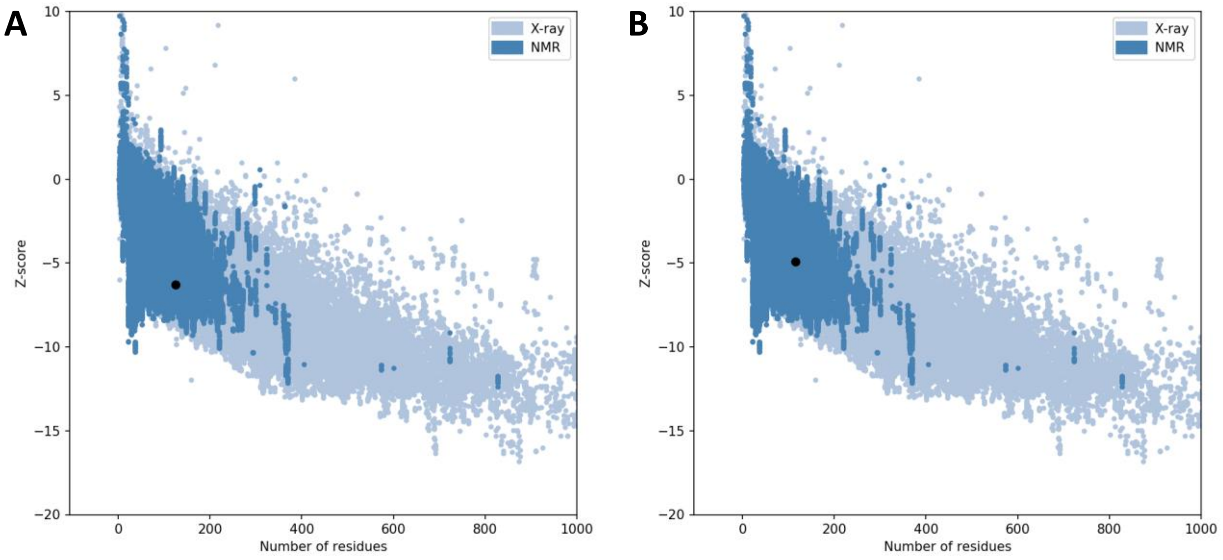

Figure S6. Prosa overall model quality plots for the *Ne*AmoD and *Ne*AmoE model structures (panels **A** and **B**, respectively).
